# A Prospective Nested Case-Control Study of Dengue in Infants: Rethinking and Refining the Antibody-Dependent Enhancement Dengue Hemorrhagic Fever Model

**DOI:** 10.1371/journal.pmed.1000171

**Published:** 2009-10-27

**Authors:** Daniel H. Libraty, Luz P. Acosta, Veronica Tallo, Edelwisa Segubre-Mercado, Analisa Bautista, James A. Potts, Richard G. Jarman, In-Kyu Yoon, Robert V. Gibbons, Job D. Brion, Rosario Z. Capeding

**Affiliations:** 1 Center for Infectious Disease and Vaccine Research, University of Massachusetts Medical School, Worcester, Massachusetts, United States; 2 Department of Immunology, Research Institute for Tropical Medicine, Manila, Philippines; 3 Department of Epidemiology, Research Institute for Tropical Medicine, Manila, Philippines; 4 Department of Molecular Biology, Research Institute for Tropical Medicine, Manila, Philippines; 5 Department of Virology, Research Institute for Tropical Medicine, Manila, Philippines; 6 Department of Virology, Armed Forces Research Institute for Medical Sciences, Bangkok, Thailand; 7 San Pablo City Health Office, San Pablo, Philippines; 8 Department of Microbiology, Research Institute for Tropical Medicine, Manila, Philippines; 9 Department of Medicine, Research Institute for Tropical Medicine, Manila, Philippines; Oxford University Clinical Research Unit, Viet Nam

## Abstract

**Background:**

Dengue hemorrhagic fever (DHF) is the severe and life-threatening syndrome that can develop after infection with any one of the four dengue virus (DENV) serotypes. DHF occurs almost exclusively in individuals with secondary heterologous DENV infections and infants with primary DENV infections born to dengue immune mothers. The widely accepted explanation for the pathogenesis of DHF in these settings, particularly during infancy, is antibody-dependent enhancement (ADE) of DENV infection.

**Methods and Findings:**

We conducted a prospective nested case-control study of DENV infections during infancy. Clinical data and blood samples were collected from 4,441 mothers and infants in up to two pre-illness study visits, and surveillance was performed for symptomatic and inapparent DENV infections. Pre-illness plasma samples were used to measure the associations between maternally derived anti-DENV3 antibody-neutralizing and -enhancing capacities at the time of DENV3 infection and development of infant DHF.

The study captured 60 infants with DENV infections across a wide spectrum of disease severity. DENV3 was the predominant serotype among the infants with symptomatic (35/40) and inapparent (15/20) DENV infections, and 59/60 infants had a primary DENV infection. The estimated in vitro anti-DENV3 neutralizing capacity at birth positively correlated with the age of symptomatic primary DENV3 illness in infants. At the time of symptomatic DENV3 infection, essentially all infants had low anti-DENV3 neutralizing activity (50% plaque reduction neutralizing titers [PRNT_50_] ≤50) and measurable DENV3 ADE activity. The infants who developed DHF did not have significantly higher frequencies or levels of DENV3 ADE activity compared to symptomatic infants without DHF. A higher weight-for-age in the first 3 mo of life and at illness presentation was associated with a greater risk for DHF from a primary DENV infection during infancy.

**Conclusions:**

This prospective nested case-control study of primarily DENV3 infections during infancy has shown that infants exhibit a full range of disease severity after primary DENV infections. The results support an initial in vivo protective role for maternally derived antibody, and suggest that a DENV3 PRNT_50_ >50 is associated with protection from symptomatic DENV3 illness. We did not find a significant association between DENV3 ADE activity at illness onset and the development of DHF compared with less severe symptomatic illness. The results of this study should encourage rethinking or refinement of the current ADE pathogenesis model for infant DHF and stimulate new directions of research into mechanisms responsible for the development of DHF during infancy.

**Trial registration:**

ClinicalTrials.gov NCT00377754

*Please see later in the article for the Editors' Summary*

## Abstract 2

Analyses of a prospective case-control study of infant dengue by Daniel Libraty and colleagues casts doubt on the antibody-dependent enhancement model for dengue hemorrhagic fever.

## Editors' Summary

**Background:**

Every year, dengue infects 50–100 million people living in tropical and subtropical areas. The four closely related viruses that cause dengue (DENV1–4) are transmitted to people through the bites of female *Aedes aegypti* mosquitoes, which acquire the viruses by feeding on the blood of an infected person. Many people who become infected with DENV have no symptoms but some develop dengue fever, a severe, flu-like illness that lasts a few days. Other people—about half a million a year—develop a potentially fatal condition called dengue hemorrhagic fever (DHF). In DHF, which can be caused by any of the DENVs, small blood vessels become leaky and friable. This leakiness causes nose and gum bleeds, bruising and, in the worst cases, failure of the circulatory system and death. There is no vaccine to prevent dengue and no specific treatment for dengue fever or DHF. However, with standard medical care—in particular, replacement of lost fluids—most people can survive DHF.

**Why Was This Study Done?:**

DHF is increasingly common, but why do only some people develop DHF after infection with DENV? The widely accepted explanation for the development of DHF is “antibody-dependent enhancement” (ADE) of DENV infection. DHF occurs almost exclusively in two settings; (i) children and adults who become infected with a second DENV serotype after an initial “primary” DENV infection with a different serotype, and (ii) infants with primary DENV infections whose mothers have some DENV immunity. The ADE model suggests that in individuals who develop DHF, although there are some antibodies (proteins made by the immune system to fight infections) against DENV in their blood (in secondary heterologous infections, antibodies left over from the primary infection; in infants with primary infections, antibodies acquired from their mothers before birth), these antibodies cannot “neutralize” the virus. Instead, they bind to it and enhance its uptake by certain immune system cells, thus increasing viral infectivity and triggering an immunological cascade that results in DHF. In this prospective, nested case-control study, the researchers directly test the ADE model for infant DHF. In a prospective study, a group of people is selected and followed to see if they develop a disease; in a nested case-control study, each case is compared with people in the group who do not develop the disease.

**What Did the Researchers Do and Find?:**

The researchers collected clinical data and blood samples from 4,441 mothers and their babies at up to two pre-illness study visits. They then followed the infants for a year to see which of them developed symptomatic and symptom-free DENV infections. Finally, they used the pre-illness blood samples to estimate the maternally derived anti-DENV antibody-neutralizing and -enhancing capacities in the infants at the time of DENV infection. 60 infants were infected with DENV—mainly DENV3—during the study. All but one infection was a primary infection. The infected infants showed a wide range of disease severity. Infants who had a high DENV3 neutralizing capacity at birth tended to develop symptomatic DENV3 infections later than infants who had a low DENV3 neutralizing capacity at birth. All the infants who developed a symptomatic DENV3 infection had a low estimated DENV3 neutralizing activity at the time of infection, and nearly all had measurable levels of DENV3 ADE activity. Infants who developed DHF did not have significantly higher frequencies or levels of DENV3 ADE activity than DENV3-infected infants with less severe symptoms.

**What Do These Findings Mean?:**

These findings indicate that maternally derived anti-DENV3 antibody initially provides protection against dengue infections. That is, babies born to DENV immune mothers are protected against dengue infections by maternally derived antibodies. Over time, the level of these antibodies declines until eventually the infant becomes susceptible to DENV infections. However, the lack of a significant association between the estimated level of DENV3 ADE activity at illness onset and the development of DHF rather than a less severe illness throws doubt onto (but does not completely rule out) the current ADE pathogenesis model for infant DHF, at least for DENV3 infections. The results of this study, the researchers conclude, should encourage rethinking or refinement of the ADE model for infant DHF and should promote further prospective studies into the development of DHF during infancy.

**Additional Information:**

Please access these Web sites via the online version of this summary at http://dx.doi.org/10.1371/journal.pmed.1000171.

- TropIKA.net provides review articles, news, opinions, research articles, and reports on dengue (in English)
- The US Centers for Disease Control and Prevention provide detailed information about dengue fever and dengue hemorrhagic fever (in English and Spanish)
- The World Health Organization provides information on dengue fever and dengue hemorrhagic fever around the world (in several languages)
- Links to additional resources about dengue are provided by MedlinePlus (in English and Spanish)
- Wikipedia has a page on antibody-dependent enhancement of viral infections (note that Wikipedia is a free online encyclopedia that anyone can edit; available in several languages)

## Introduction

Dengue hemorrhagic fever (DHF) is the most severe and sometimes fatal form of illness after infection with any one of the four dengue virus (DENV) serotypes [1]. The global spread of dengue, and the incidence of epidemic DHF, have increased dramatically over the past 50 y and continue on an upward trajectory [2],[3]. An accurate understanding of DHF pathogenesis is important for clinicians, public health officials, and vaccine researchers in dengue affected countries. DHF occurs almost exclusively in two clinical settings: children and adults with secondary heterologous DENV infections and infants with primary DENV infections born to dengue-immune mothers [3]. The most widely accepted and repeatedly cited explanation for the pathogenesis of DHF in these settings is antibody-dependent enhancement (ADE) of DENV infection [4]–[6]. The ability of subneutralizing virus-specific antibodies to enhance DENV as well as other flavivirus infections in vitro was first recognized in the 1960s [7] and has been extensively studied [8]–[11]. The ADE model for DHF proposes that anti-DENV IgG, actively acquired from a previous heterologous DENV infection, or passively acquired in infants from maternal-fetal transfer, enhances DENV infection of Fc receptor-bearing cells under particular conditions in vivo. The ADE of DENV infection increases viral load (infected cell mass) and triggers a host immunological cascade that leads to DHF [3],[12].

The lynchpin of the ADE disease model lies in its explanation of DHF in infants, where maternally derived anti-DENV IgG levels decay over time [13] in the absence of virus-specific memory B- and T cells. The argument for a pathogenic and central role of ADE in infant DHF has been largely based on linking two epidemiological associations. In dengue endemic regions, the age-related prevalence of infant primary DHF peaks around 6–8 mo of age and then drops off to low levels [12],[14],[15]. In blood samples collected from infant cohorts in dengue endemic regions, this age range generally coincides with peak in vitro ADE activity [12],[14],[16]. We are conducting a prospective clinical study in the Philippines of DENV infections during infancy. We have performed a nested case-control study of infant DENV infections during the 2007 season to directly measure the associations between maternally derived anti-DENV IgG-neutralizing and -enhancing capacities at the time of infection and development of DHF. This report is the first to directly test predictions of the ADE model for infant DHF by using pre-illness plasma samples collected from infants with primary DENV infections and a wide spectrum of clinical disease severity. The results provide important new information regarding infant dengue and the development of DHF.

## Methods

### Ethics Statement

The study protocol was approved by the institutional review boards of the Research Institute for Tropical Medicine, Philippines, and the University of Massachusetts Medical School. Mothers and their healthy infants were recruited and enrolled after providing written informed consent. The clinical study is registered at www.clinicaltrials.gov (identifier NCT00377754), and the study protocol is provided as supporting information (Text S1).

### Study Design

Study enrollment began in October 2006, and surveillance for acute febrile illnesses began in January 2007, in San Pablo, Laguna, Philippines. This paper describes cases identified between January 2007 and January 2008 and is the first report from the ongoing clinical study.

**Figure 1:**
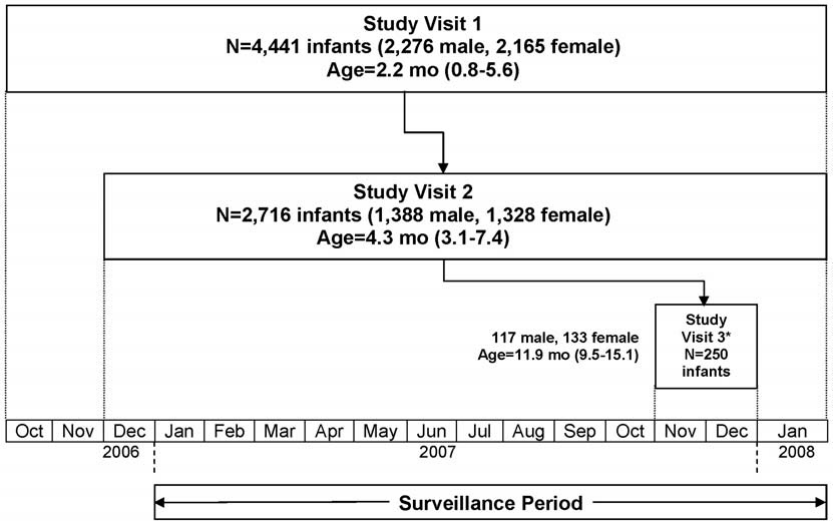
Flowchart of study participation, October 2006–January 2008. The ages at each study visit are presented as the median (range) in months. *, A subset of 250 infants who had completed study visits 1 and 2 were selected for a third study visit in November/December 2007. These infants did not have any previously reported febrile illnesses.

Blood samples were collected from the infant and mother at the first study visit when the infant was approximately 6–18 wk old. A second serial blood sample was collected from infants at their next study visit between ages 4–7 mo. A third serial blood sample was collected in November/December 2007 from a subset of 250 infants without any reported febrile illnesses (Figure 1). Clinical and epidemiological information were also collected at each study visit. Normalized child growth indicators were determined using World Health Organization (WHO) child growth standards [17]. We conducted surveillance year round for hospitalized acute febrile illnesses in study infants across the seven hospitals serving San Pablo. During the rainy season (June–November 2007), mothers were encouraged to bring their infants to the San Pablo City Health Office for evaluation of outpatient febrile illnesses. Acute- and convalescent-phase (day 14) blood samples were obtained from study infants with febrile illnesses that did not have an obvious source at time of presentation (e.g., lobar pneumonia, bacterial meningitis, pyelonephritis). Routine clinical information was abstracted daily during any hospitalization and at the acute and convalescent time points for all febrile study infants.

### Identification and Characterization of DENV Infections

A DENV infection was identified in febrile infants by serotype-specific reverse-transcription (RT)-PCR in acute-phase sera [18] and DENV IgM/IgG ELISA [19] in paired acute and convalescent phase sera. Primary or secondary DENV infections were identified by previously established serologic criteria for the paired IgM/IgG ELISA results [19]. The infecting DENV serotype was identified by RT-PCR for all the symptomatic infants except one. A sample for RT-PCR was not collected for this infant, but a monotypic rise in anti-DENV3 neutralizing antibody titers was seen in the paired acute and convalescent phase sera. Viremia levels in acute-phase sera from DENV3-infected infants were quantified using a validated qRT-PCR assay [20].

Serial blood samples (study visits 1, 2, and 3) from a subset of 250 infants without reported febrile illnesses were screened for evidence of clinically inapparent DENV infection using a hemagglutination-inhibition (HAI) assay to DENV1-4 and Japanese encephalitis virus (JEV) [21]. Infants with DENV/JEV HAI titers ≤40 and <4-fold changes across study visits 1, 2, and 3 were identified as DENV-uninfected during the surveillance period (nondengue controls). Infants with a ≥4-fold rise in DENV HAI titers between two time points were then tested by plaque reduction neutralizing antibody assay to DENV1-4 and JEV, as described below. A primary DENV infection was identified by a >4-fold rise in DENV 50% plaque reduction neutralization titers (PRNT_50_) between two time points with a monotypic pattern [22]. The DENV serotype with the highest PRNT_50_ in a monotypic pattern was assumed to be the serotype that produced the clinically inapparent infection.

Laboratory and radiographic investigations for hospitalized infants with DENV infections were directed by the treating physicians. There were ≥3 serial determinations of hematocrit and platelets performed for all the hospitalized infants and covering the period of defervescence. Hemoconcentration was measured by comparing the maximum recorded hematocrit (around the time of defervescence and platelet nadir) with the minimum recorded hematocrit at either the beginning or end of hospitalization. None of the infants received red blood cell or whole blood transfusions. Hospitalized infants with DENV infection were classified as having DHF only when review of their clinical course and all clinical data strictly met the WHO classification criteria [23]. All the nonhospitalized infants with DENV infections had mild self-resolving febrile illnesses <1 wk in duration.

### Antibody-Mediated Neutralization and Enhancement Assays

Plaque reduction neutralizing antibody assays against DENV3 strain 16562 were performed on serial dilutions of heat-inactivated (56°C×30 min) maternal and pre-illness plasma samples, as previously described [22]. DENV3 PRNT_50_ were determined using a sigmoid dose-response (variable slope) curve fit and reported as reciprocal values. The maternal plasma PRNT_50_ was used to estimate the PRNT_50_ at birth in the infant. Only one mother had evidence of a DENV infection between infant birth and the first study visit and was excluded from data analysis. The mother did not report a febrile illness between birth and the first study visit when her infant was 63 d old. However, concurrent maternal and infant DENV3 PRNT_50_ at the first study visit were 10,762 and 20, respectively.

ADE experiments were performed with FcγIIa receptor-bearing K562 cells [10] under previously optimized conditions. In brief, the most proximal pre-illness plasma sample from each infant was heat inactivated (56°C×30 min) and diluted to achieve the neutralizing capacity at time of infection. This plasma dilution was pre-incubated with DENV3 strain 16562 for 1 h at 37°C and then added to 2.5×10^5^ K562 cells at a multiplicity of infection = 0.015. Cell culture supernatants were collected at 72 h and DENV3 levels measured by qRT-PCR assay (values expressed as DENV3 genome equivalents [eqs]/ml) [20]. Three independent ADE experiments were performed. Negative controls were DENV3 infection alone and DENV3 pre-incubated with flavivirus seronegative plasma. The positive control was DENV3 pre-incubated with a dilution of DENV3-immune infant plasma.

### Statistical Analysis

SPSS (version 12.0) and STATA (version 9.0) software were used for the statistical analyses. SigmaPlot (version 9.0) was used to fit plaque reduction neutralization data to sigmoid dose-response (variable slope) curves [11] and determine the PRNT_50_. Parametric tests (Student's *t*-test, ANOVA, Pearson correlation) were used for comparisons and correlations among normally distributed variables. Wilcoxon ranked-sum and Kruskal-Wallis tests were used for comparisons of variables that were not normally distributed. Spearman's rank correlation was used for correlations between variables not normally distributed. Pearson χ^2^ test was used for comparisons of categorical variables among disease severity groups. A mixed-effects least-squares regression model with compound symmetry was used for comparisons of the ADE experimental data with three repeated measures (*p*-values were obtained from the model). Mean or median values are presented with their respective 95% confidence intervals (CIs). *p*<0.05 was considered significant; 0.05≤ *p*-values <0.10 were considered a nonsignificant trend.

## Results

### Study Participants and Characteristics

4,441 infants and their mothers participated in the prospective study of DENV infections during the time period covered in this report (Figure 1). Surveillance between January 2007 and January 2008 captured 97 hospitalized and 256 nonhospitalized infants with acute febrile illnesses that did not have an obvious source at the time of presentation. 40/353 (11%) of these acute febrile illnesses were caused by DENV infections. In a subset of 250 infants without reported febrile illnesses through November/December 2007, we identified an additional 20 infants (8%) who had a clinically inapparent DENV infection. The characteristics of the DENV-infected infants are shown in Table 1. DENV3 was the predominant serotype among the infants with symptomatic (35/40) and inapparent (15/20) DENV infections in the 2007 season. 59/60 infants had a primary DENV infection, and subsequent analyses excluded the one infant with a secondary DENV3 infection. There was a 1∶1 ratio of hospitalized∶nonhospitalized illnesses among infants with symptomatic primary dengue. 9/20 (45%) of the infants with hospitalized primary dengue had unambiguous DHF. Five of these infants had DHF grades III/IV (dengue shock syndrome [DSS]), including one infant who died. The remaining four infants were classified as DHF grade I with hemoconcentration ranging from 27%–44% (one DHF grade I infant also had a large pleural effusion on a chest radiograph). None of the DENV-infected infants had clinically significant bleeding.

**Table 1:** Study subject characteristics.

Study Subject Characteristics	Symptomatic DENV Infection^a^ (*n* = 40)			Inapparent DENV Infection^a^ (*n* = 20)	Comparisons (*p*-values)
	Hospitalized (*n* = 21)		Not Hospitalized (*n* = 19)		
	DHF (*n* = 9)	Not DHF (*n* = 12)	Not DHF (*n* = 19)		
**DENV serotype**	DENV3 (*n* = 9)	DENV3 (*n* = 12)	DENV1 (*n* = 1); DENV2 (*n* = 4); DENV3 (*n* = 14)	DENV2 (*n* = 5); DENV3 (*n* = 15)	—
**1° versus 2° DENV infection**	1°−*n* = 9	1°−*n* = 11; 2°−*n* = 1	1°−*n* = 19	1°−*n* = 20	—
**Gender ratio (male∶female)** b	6∶3	7∶4	11∶8	10∶10	*p* = 0.9
**Age at onset of dengue illness (mo)** b **(median, 95% CI)**	5.7 (4.3–6.9)	^c^	^c^	—	*p* = 0.09 (DHF versus symptomatic, not DHF)
**Age at study enrollment (mo)** b **(median, 95% CI)**	2.5 (1.6–3.5)	^d^	^d^	2.3 (2.0–2.4)	*p* = 0.9

a Infants with symptomatic and inapparent DENV infections were identified as described in Methods.

b Infant with secondary DENV infection excluded (primary DENV infections only).

c 7.9 (6.5-9.3) for these two categories combined.

d 2.4 (1.8-4.6) for these two categories combined.

**Figure 2:**
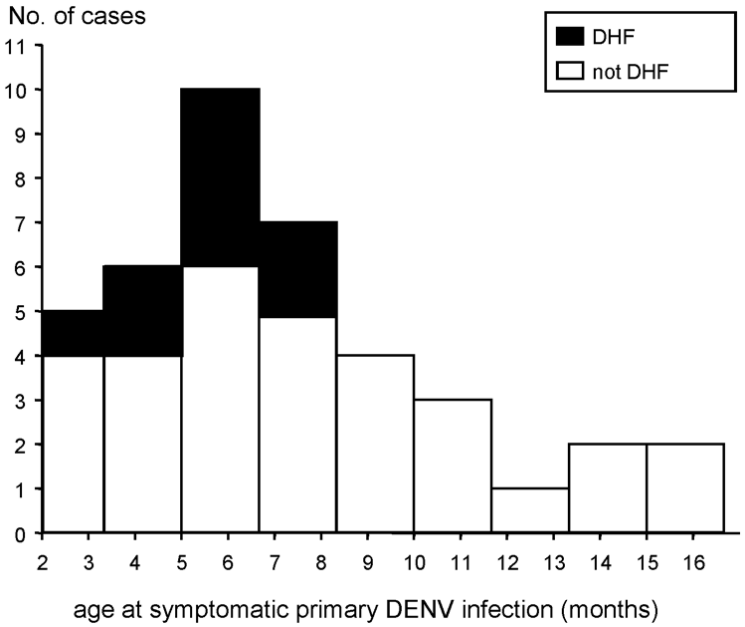
Age distribution of infants with symptomatic primary DENV infections. Filled bars, hospitalized infants with DHF. Open bars, hospitalized and nonhospitalized infants without DHF.

**Figure 3:**
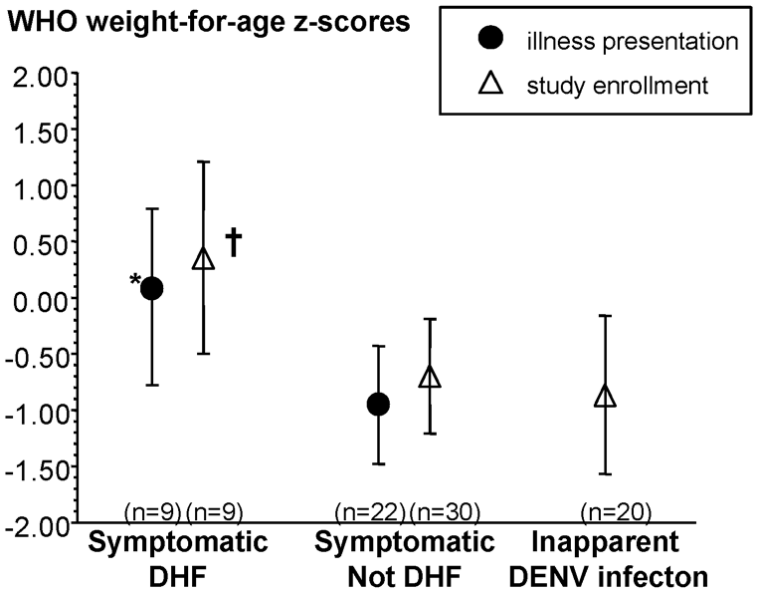
World Health Organization (WHO) weight-for-age z-scores at study enrollment and acute illness in infants with primary DENV infections. Closed circles, weight-for-age z-scores at illness presentation; open triangles, weight-for-age z-scores at study enrollment. Values are presented as the mean with 95% CIs. *, *p* = 0.04, weight-for-age z-scores at presentation in DHF infants versus symptomatic dengue, not DHF, infants. †, *p* = 0.03, weight-for-age z-scores at study enrollment in DHF infants versus symptomatic and inapparent dengue, not DHF, infants.

All the infants included in the nested case-control analysis were born at full-term by maternal history. The median age at study enrollment was 2.4 mo (Table 1). The peak number of symptomatic primary DENV infections was seen between ages 4–8 mo (Figure 2). There was a nonsignificant trend towards a younger age at time of illness in the infants with DHF compared to symptomatic dengue without DHF (median age at illness onset 5.7 versus 7.9 mo, DHF versus not DHF, *p* = 0.09). The DHF infants had higher weight-for-age z-scores [17] on illness presentation compared to the symptomatic infants without DHF (*p* = 0.04). A higher weight-for-age z-score at study enrollment was also associated with subsequent development of DHF compared to all other DENV-infected infants (*p* = 0.03) (Figure 3). Length-for-age z-scores also trended higher in the DHF infants at study enrollment (*p* = 0.07), but were not measured at illness presentation. Weight-for-length z-scores were not different between DHF versus not DHF infants at study enrollment (*p* = 0.4).

### Antibody-Mediated Neutralizing Capacity at the Time of Primary DENV3 Infection

In order to examine the potential role of maternally derived anti-DENV IgG in shaping disease severity, we estimated the DENV3 PRNT_50_ at time of infection in the 34 infants with symptomatic primary DENV3 infections. We calculated individual first-order decay rates of maternally derived DENV3 PRNT_50_ in these 34 infants using two infant pre-illness plasma samples (*n* = 17), or one maternal and one infant pre-illness plasma sample (*n* = 14) (median decay rate was used in *n* = 3 infants). Endpoint DENV3 PRNT_50_ at the time of infection were then extrapolated. In these infants and DENV-uninfected controls (*n* = 30), the mean half-life (t_1/2_) of DENV3 PRNT_50_ was 38 d (95% CI 34–42 d), and was not significantly different if calculated using two infant plasma samples or one maternal and one infant plasma sample (*p* = 0.3).

**Figure 4:**
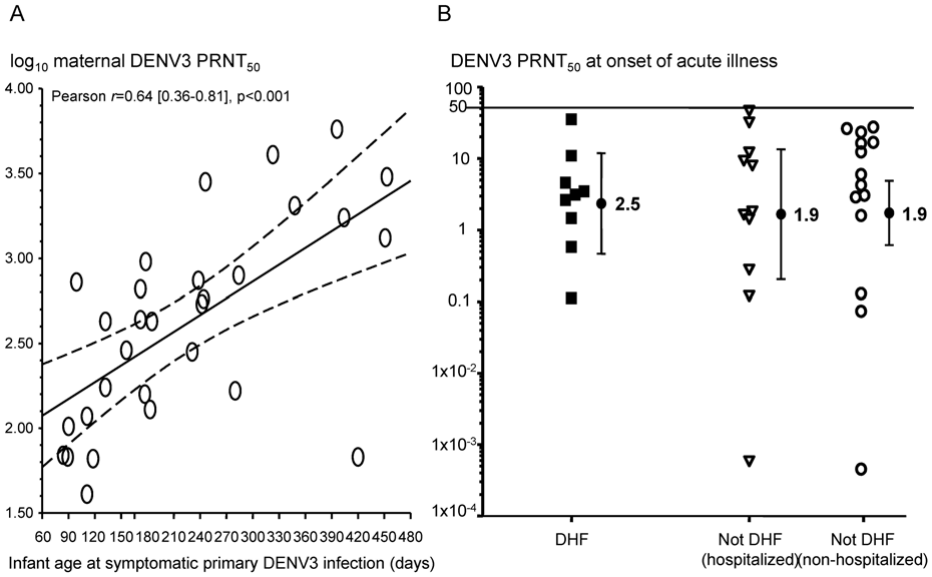
Estimated DENV3 neutralizing capacities at birth and illness onset in infants with symptomatic primary DENV3 infections. (A) Log_10_ transformed PRNT_50_ to DENV3 in maternal plasma (estimate for birth time point) were positively correlated with infant age at onset of symptomatic primary DENV3 infection (*n* = 30). Mean (95% CI) of the Pearson correlation coefficient (*r*) is shown. The linear regression curve and 95% CI are shown as a solid line and dashed lines, respectively. (B) Endpoint DENV3 PRNT_50_ at the time of symptomatic primary DENV3 infection were extrapolated from pre-illness plasma samples as described in the Results (*n* = 34). Closed squares, hospitalized infants with DHF; open triangles, hospitalized infants without DHF; open circles, nonhospitalized infants without DHF. The closed circles and error bars are the DENV3 PRNT_50_ geometric mean titers (GMT) and 95% CI, respectively. There were no significant differences among the disease severity groups (*p* = 0.9).

A protective role for maternally derived anti-DENV3 IgG was suggested by a strong positive correlation between the estimated DENV3 PRNT_50_ at birth and the infant age at onset of symptomatic primary DENV3 infection (Pearson *r* = 0.64 [0.36–0.81], *p*<0.001) (Figure 4A). At the time of symptomatic infection, extrapolated DENV3 PRNT_50_ were ≤50 in all infants and the geometric mean titer (GMT) was <5. There were no significant associations between the extrapolated DENV3 PRNT_50_ at time of symptomatic infection and disease severity (*p* = 0.9) (Figure 4B).

### Antibody-Mediated Enhancing Capacity at the Time of Primary DENV3 Infection

**Figure 5:**
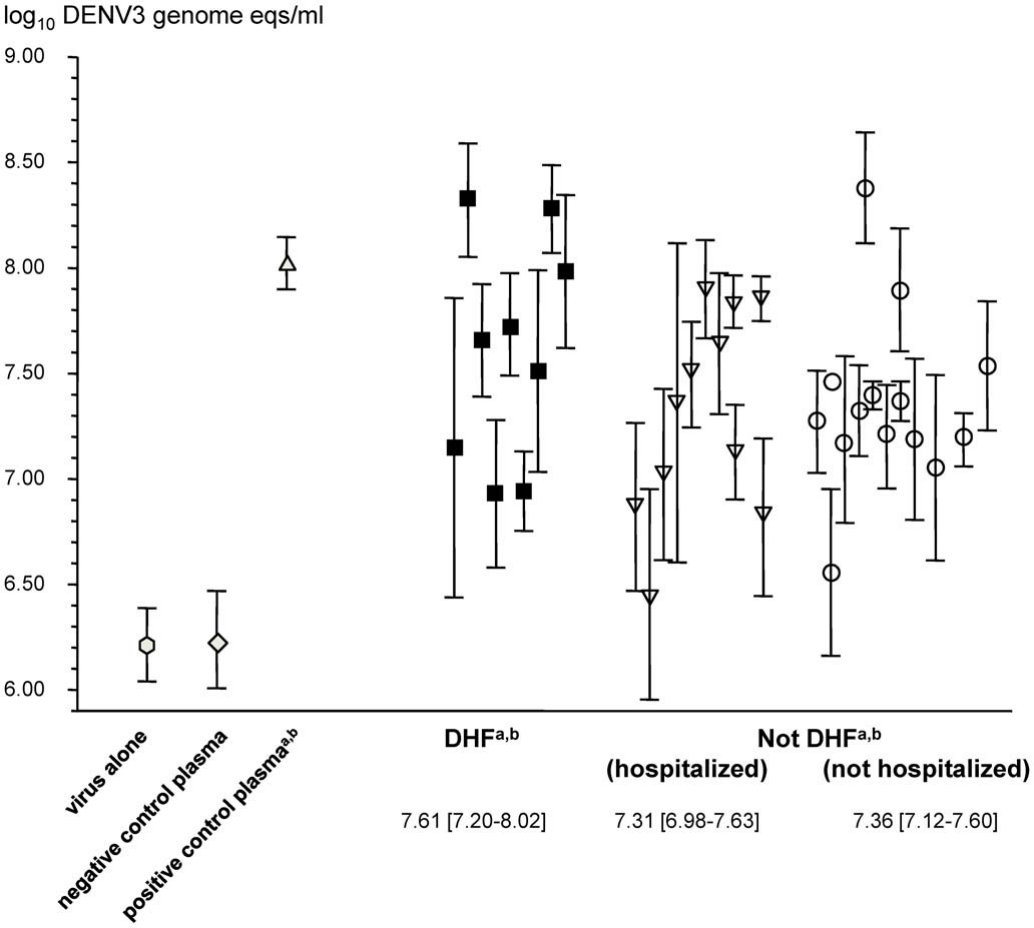
ADE of DENV3 infection at illness onset in infants with symptomatic primary DENV3 infections. The most proximal pre-illness plasma sample from each infant with symptomatic primary DENV3 infection (*n* = 34) was diluted to achieve the estimated neutralizing capacity at illness onset and used in the ADE assay, as described in the Methods. Values are log_10_ transformed DENV3 genome eqs/ml in cell culture supernatants at 72 h, mean±standard deviation of individual plasma samples from three independent experiments. Mean DENV3 genome eqs/ml and 95% CI are shown for each of the disease severity groups (mean [95% CI]). Closed squares, hospitalized infants with DHF; open triangles, hospitalized infants without DHF; open circles, nonhospitalized infants without DHF. ^a^ *p*<0.01 compared to virus alone; ^b^ *p*<0.01 compared to flavivirus seronegative plasma control. There were no significant differences among the disease severity groups (see Results).

**Figure 6:**
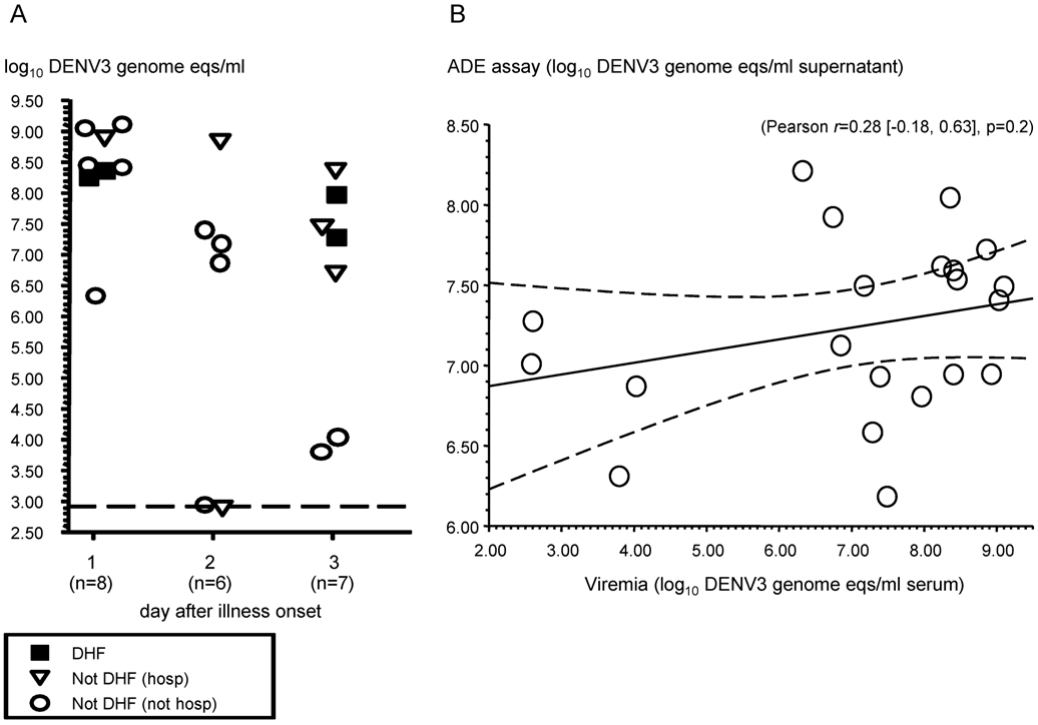
Early viremia levels and ADE activity at illness onset in infants with symptomatic primary DENV3 infections. (A) Early viremia levels in acute-phase sera from infants with symptomatic primary DENV3 infections. Values are log_10_ transformed DENV3 genome eqs/ml measured in single acute-phase serum samples collected between 1–3 d after illness onset. Closed squares, hospitalized infants with DHF; open triangles, hospitalized infants without DHF; open circles, nonhospitalized infants without DHF. Line represents the lower limit of quantitation (2.93 log_10_ DENV3 genome eqs/ml). (B) Mean log_10_ transformed DENV3 genome eqs/ml from ADE assay cell culture supernatants versus viremia levels in single acute-phase serum samples collected between 1–3 d after illness onset (*n* = 21). Mean [95% CI] of the Pearson correlation coefficient (*r*) is shown. The linear regression curve and 95% CI are shown as a solid line and dashed lines, respectively.

The ADE model predicts that infants who develop DHF will have maternally derived anti-DENV IgG levels at the time of infection that enhance DENV infection to a greater degree than infants without DHF. We diluted the most proximal pre-illness plasma sample from the 34 infants with symptomatic primary DENV3 infection to achieve their estimated DENV3 PRNT_50_ at illness onset and measured the concomitant ADE activity. In nearly all cases, pre-incubation of DENV3 with the plasma dilutions increased viral production from K562 cells compared with virus alone or flavivirus seronegative plasma. The plasma dilutions from 9/9 infants with DHF and 22/25 infants without DHF were able to increase DENV3 production >0.5 log_10_ genome eqs/ml above virus infection alone (*p* = 0.6). There were no significant associations between measures of disease severity and antibody-mediated DENV3 enhancing capacity at the time of infection (DHF versus not DHF, *p* = 0.13; DHF versus not hospitalized, not DHF, *p* = 0.19; hospitalized versus not hospitalized, *p* = 0.5) (Figure 5). The ADE pathogenesis model also predicts that enhancing capacity at the time of DENV infection should positively correlate with viral load. We measured viremia levels by qRT-PCR in single acute illness plasma samples from 33/34 infants with symptomatic primary DENV3 infections. As expected, the viremia levels were inversely correlated with the day of illness (Spearman's *r* = −0.62, *p*<0.001). We used viremia levels obtained within 3 d after illness onset as the best available estimate of peak viremia [24],[25]. These early viremia levels ranged from 2.9–9.1 log_10_ DENV3 genome eqs/ml (Figure 6A). During this time period, there was a nonsignificant trend towards higher mean viremia levels in infants with DHF compared to those without DHF (DHF versus not DHF, 8.0±0.5 versus 6.9±2.2 log_10_ DENV3 genome eqs/ml [mean ± standard deviation], *p* = 0.07). Regardless of disease severity classification, the DENV3 enhancing capacity at time of infection did not correlate with the early viremia levels (Pearson *r* = 0.28 [−0.18, 0.63], *p* = 0.2) (mean regression slope [95% CI], 0.07 [−0.05 to 0.19] (Figure 6B).

## Discussion

This prospective study has captured the entire spectrum of clinical disease severity among infants with primary DENV infections—ranging from inapparent infections, mild outpatient febrile illnesses, hospitalized illnesses without evidence of DHF, and unambiguous hospitalized DHF/DSS. DENV3 was the predominant infecting serotype in the 2007 dengue season covered by this report. We therefore focused on the potential role of maternally derived anti-DENV IgG in shaping DENV3 disease severity. Our data support an initial in vivo protective role for high levels of maternally derived anti-DENV3 IgG at birth. The estimated in vitro anti-DENV3 neutralizing capacity at birth (maternal DENV3 PRNT_50_ geometric mean titer [GMT] = 413, range 41–5,690) positively correlated with the infant age of symptomatic primary DENV3 illness (all disease severity groups). A similar correlation was previously reported for estimated anti-DENV2 neutralizing capacity at birth in 13 DHF infants with primary DENV2 infections [16]. A more recent study did not report a correlation between estimated serotype-specific neutralizing capacity at birth and the infant age at dengue illness [26]. However, this study combined serotype-specific PRNT_50_ values for infants with DENV1-3 infections in their analysis and performed a different in vitro neutralization assay.

All the infants in our study with symptomatic DENV3 infections had a DENV3 PRNT_50_ ≤50 at the time of infection, and there were no correlations between the neutralizing capacity at time of infection and DENV3 disease severity. These data support that primary DENV3 febrile illnesses occurred in infants when maternally derived anti-DENV3 IgG were below effective in vivo neutralizing concentrations. Identifying levels of neutralizing antibody associated with clinical protection is an important issue for dengue vaccine development and testing, as such correlates of protective immunity have not been previously well defined. The estimated neutralizing antibody titers at the time of primary DENV infection in infants represent a natural study of passive immunization. For DENV3 infections, our results suggest that a DENV3 PRNT_50_ >50 is likely to correlate with clinical protection but a measurable DENV3 PRNT_50_ ≥10 and ≤50 is not. These findings are remarkably consistent with an earlier study we conducted in older children with secondary DENV3 infections [22]. Using the same DENV3 strain in the neutralization assays, we reported that only a pre-illness DENV3 PRNT_50_≥100 was associated with lower viremia and milder DENV3 disease severity. We anticipate that similar neutralizing antibody data for other DENV serotypes will emerge as our infant clinical study continues.

The current ADE model predicts that infant DHF develops when subneutralizing maternally derived anti-DENV IgG enhances DENV infection in Fc receptor-bearing cells to higher levels than in infants with subneutralizing anti-DENV IgG levels who do not develop DHF [12]. We found that essentially all infants with symptomatic DENV3 infections had subneutralizing plasma IgG levels and measurable Fc receptor-dependent DENV3 ADE activity at the time of infection. The infants who developed DHF did not have significantly higher frequencies or levels of DENV3 ADE activity compared to symptomatic infants without DHF. We recognize that some hospitalized infants classified as “not DHF” may have met the WHO criteria for DHF with more intensive investigations. However, there were no significant differences in DENV3 ADE activity between the most severe hospitalized DENV3 illnesses and mild outpatient DENV3 illnesses, or between all hospitalized and nonhospitalized infants. Our data cannot exclude that mean ADE levels in symptomatic infants without DHF were actually ≤1.0 log_10_ DENV3 genome eqs/ml lower than in the DHF infants. Notably, ADE and early viremia levels varied widely across all the symptomatic DENV3-infected infants, even among those with mild symptomatic primary DENV3 illnesses. DENV3 ADE activity at the time of infection and early viremia levels were also not correlated, although a significant positive association with a regression slope <0.2 may have been missed. Our prospective study is the first to directly examine ADE activity at the time of infection among infants exhibiting a wide spectrum of dengue disease severity. The results suggest that measurable ADE activity is common and varied across all symptomatic DENV3-infected infants, and no significant associations with DHF have yet emerged. There has been only one previous study that examined and reported a direct association between ADE activity and infants with primary DHF [16]. The authors reported that peak DENV2 ADE activity in 5/13 diluted sera from mothers of infants with primary DENV2 DHF were above a cutoff value compared to 2/22 sera from mothers of infants with nondengue febrile illnesses and toddlers with secondary DHF. The dilution of maternal sera that produced peak ADE activity was not directly linked to the time of DENV2 infection in the DHF infants. The reported data were from a single experiment, and there was no comparison to primary DENV2-infected infants without DHF.

We measured Fc receptor-dependent ADE of DENV3 infection using the K562 human cell line and a highly reproducible DENV3 qRT-PCR assay. Experimental conditions for ADE assays have not been standardized and have been previously debated [27]. The K562 cell line has been used by others to reliably measure ADE of DENV infection in vitro [10],[28],[29] and support the temporal association between ADE activity and infant DHF [14]. DENV qRT-PCR is a sensitive, accurate, and reproducible method to measure viral production, and has always correlated with plaque-forming virus titers or percentage of infected cells in ADE assays [10],[30]. The ADE results and conclusions in this report are restricted to the DENV3 serotype, though the ADE model has been proposed as a general mechanism for all infant DHF. Future data from the ongoing clinical study should help to determine whether the current lack of a positive correlation between ADE activity and symptomatic dengue disease severity will hold up within narrower confidence intervals, and also extend our findings beyond primary infant DENV3 infections.

There is the possibility that ADE of DENV infection is important in the pathogenesis of all symptomatic primary infant dengue (including mild, outpatient febrile illnesses) compared to inapparent/asymptomatic dengue. In Figure 4A, the observed infant ages may be clustered in a window period after maternally derived anti-DENV3 IgG drop below effective in vivo neutralizing concentrations. If inapparent DENV3 infections are not similarly clustered (i.e., shifted to the right of the symptomatic DENV3 infections), then it would suggest that an antibody-mediated effect is associated with symptomatic DENV3 illness. We could not pinpoint the time of inapparent DENV3 infections and therefore could not include this group in Figure 4A or assess their relevant ADE activity. Additional studies that can accurately capture the ages of inapparent infant DENV infections will be important in this regard.

Interestingly, we found that a higher weight-for-age z-score in the first few months of life was a risk factor for developing DHF from a subsequent primary DENV infection during infancy. This association persisted at the time of illness presentation among infants with symptomatic primary DENV infections. Some earlier studies have suggested that infants with DHF are less likely to be malnourished at presentation compared to healthy or nondengue illness controls [31],[32]. A younger infant age at the time of a primary DENV infection has also been previously reported as a risk factor for DHF [12]. Our study demonstrated this trend but notably it was independent of ADE activity. The anthropometric and age-related observations in this study should stimulate investigations into novel potential host mechanisms involved in the pathogenesis of infant DHF. These observations should also alert clinicians caring for infants in dengue-endemic countries. A high index of suspicion and close monitoring for DHF should take place particularly for febrile infants ≤8 mo old with above average WHO weight-for-age z-scores for their population.

In summary, our prospective nested case-control study found that DENV-infected infants exhibited a wide range of disease severity. We identified levels of maternally derived neutralizing antibody associated with clinical protection against symptomatic DENV3 illness. Measurable ADE activity at illness onset and early viremia levels varied widely across all symptomatic DENV3-infected infants, including those with mild outpatient illnesses. The results should encourage rethinking or refinement of the currently promulgated ADE model for infant DHF, promote prospective studies of infant dengue, and stimulate new directions of research into novel potential mechanisms for infant DHF.

## Supporting Information

Text S1 Protocol.
(0.15 MB PDF)

## Abbreviations

ADE: antibody-dependent enhancement
CI: confidence interval
DENV: dengue virus
DHF: dengue hemorrhagic fever
eqs: equivalents
HAI: hemagglutination-inhibition
PRNT_50_: 50% plaque reduction neutralization titers
RT: reverse transcription
