# Supplementary material for: A Prospective Nested Case-Control Study of Dengue in Infants: Rethinking and Refining the Antibody-Dependent Enhancement Dengue Hemorrhagic Fever Model
Source: PLoS Med. 2009 Oct 27;6(10):e1000171. doi: 10.1371/journal.pmed.1000171 (PMC2762316; doi:10.1371/journal.pmed.1000171)
Supplement: Text S1 — Protocol. (0.15 MB PDF) [file pmed.1000171.s001.pdf]

TITLE

A Prospective Study of Dengue Virus Infections during Infancy to Define Correlates of Protective Immunity

DMID Protocol Number: 06-0013

Sponsored by:
National Institute of Allergy and Infectious Diseases (NIAID)

DMID Funding Mechanism:
U01-AI-065654-01

Principal Investigator:
Daniel H. Libraty, M.D.

DMID Protocol Champion:
Walla Dempsey, PhD.

Draft or Version Number:
3.0

Day Month Year
28 March 2006

Statement of Compliance

The study will be carried out in accordance with Good Clinical Practice as required by the Edinburgh revision (October 2000) of the Declaration of Helsinki as far as adopted by the concerned regulatory authorities, as well as International Conference on Harmonisation, Good Clinical Practice E6, and the National Institutes of Health (NIH) Clinical Terms of Award. The study will also be conducted in accordance with the applicable national and local requirements regarding ethical committee review, informed consent, and other statutes or regulations regarding the protection of the rights and welfare of human subjects participating in biomedical research.

SIGNATURE PAGE

The signatures below document the approval of this protocol and the attachments, and provide the necessary assurances that this clinical study will be conducted according to all stipulations of the protocol, including all statements regarding confidentiality and according to local legal and regulatory requirements and to the principles outlined in applicable U.S. federal regulations and International Conference on Harmonisation guidelines.

Principal Investigator – *Name of Site*:
University of Massachusetts Medical School
Signed: ____________________ Date: 07/24/06

Name: Daniel H. Libraty
Title: Associate Professor of Medicine

Table of Contents

Statement of Compliance i
Signature Page ii
List of Abbreviations iv
Protocol Summary v
1 Key Roles 1
2 Background Information and Scientific Rationale 3
2.1 Background Information 3
2.2 Scientific Rationale 5
2.3 Potential Risks and Benefits 6
2.3.1 Potential Risks 6
2.3.2 Known Potential Benefits 6
3 Objectives 7
4 Study Design 8
5 Study Population 14
5.1 Selection of the Study Population 14
5.2 Inclusion/Exclusion Criteria 14
6 STUDY PROCEDURES/EVALUATIONS 15
6.1 Study Procedures 15
6.2 Laboratory Evaluations 16
6.2.1 Laboratory Evaluations/Assays 16
6.2.2 Specimen Collection, Preparation, Handling and Shipping 19
7 Statistical Considerations 20
7.1 Study Outcome Measures 20
7.2 Sample Size Considerations 20
7.3 Participant Enrollment and Follow-Up 21
7.4 Analysis Plan 21
8 Subject Confidentiality 23
8.1 Future Use of Stored Specimens 23
9 Informed Consent Process 25
9.1 Informed Consent/Assent Process (in Case of a Minor or Others Unable to Consent for Themselves) 25
10 Literature References 26

SUPPLEMENTS/APPENDICES

A. Informed consent form for the infants

B. Informed consent form for mothers.

C. Advertisement sheet

D. Study information for recruitment sessions

List of Abbreviations

Ab	Antibody
ADE	Ab dependent enhancement
CIDVR	Center for Infectious Disease and Vaccine Research, UMMS
CRF	Case Report Form
DF	Dengue fever
DHF	Dengue hemorrhagic fever
DMID	Division of Microbiology and Infectious Diseases, NIAID, NIH, DHHS
DV	Dengue virus
EIA	Enzyme linked immunosorbent assay
EPI	Extended Programme in Immunization
HAI	Hemagglutination inhibition
N	Number (typically refers to subjects)
NIAID	National Institute of Allergy and Infectious Diseases, NIH, DHHS
NIH	National Institutes of Health
PBMC	Peripheral blood mononuclear cells
QA	Quality assurance
RITM	Research Institute of Tropical Medicine, Department of Health, Manila, Philippines
RT-PCR	Reverse transcriptase-polymerase chain reaction
UMMS	University of Massachusetts Medical School
WHO	World Health Organization

Protocol Summary

Title: A Prospective Study of Dengue Virus Infections during Infancy to Define Correlates of Protective Immunity.

Population: Healthy infants between the ages of 6 weeks-16 months and their mothers in the San Pablo Health District, Laguna, Philippines.

Number of subjects to be enrolled: 10,000 mother-infant pairs.

Number of Sites: 7 sites, San Pablo Health District, Laguna, Philippines

Study Duration: 4 years

Subject Duration: 50-58 weeks

Objectives:

Primary:

- Define the levels of serotype-specific neutralizing antibody (Ab) associated with protective immunity against symptomatic dengue.

Secondary:

- Delineate specific viral, infant, and maternal factors contributing to the development of dengue hemorrhagic fever (DHF) among infants experiencing primary dengue virus (DV) infections.
- Strengthen the capacity for diagnosis and research on dengue in the Philippines.

Schematic of Study Design:

(see page vi)

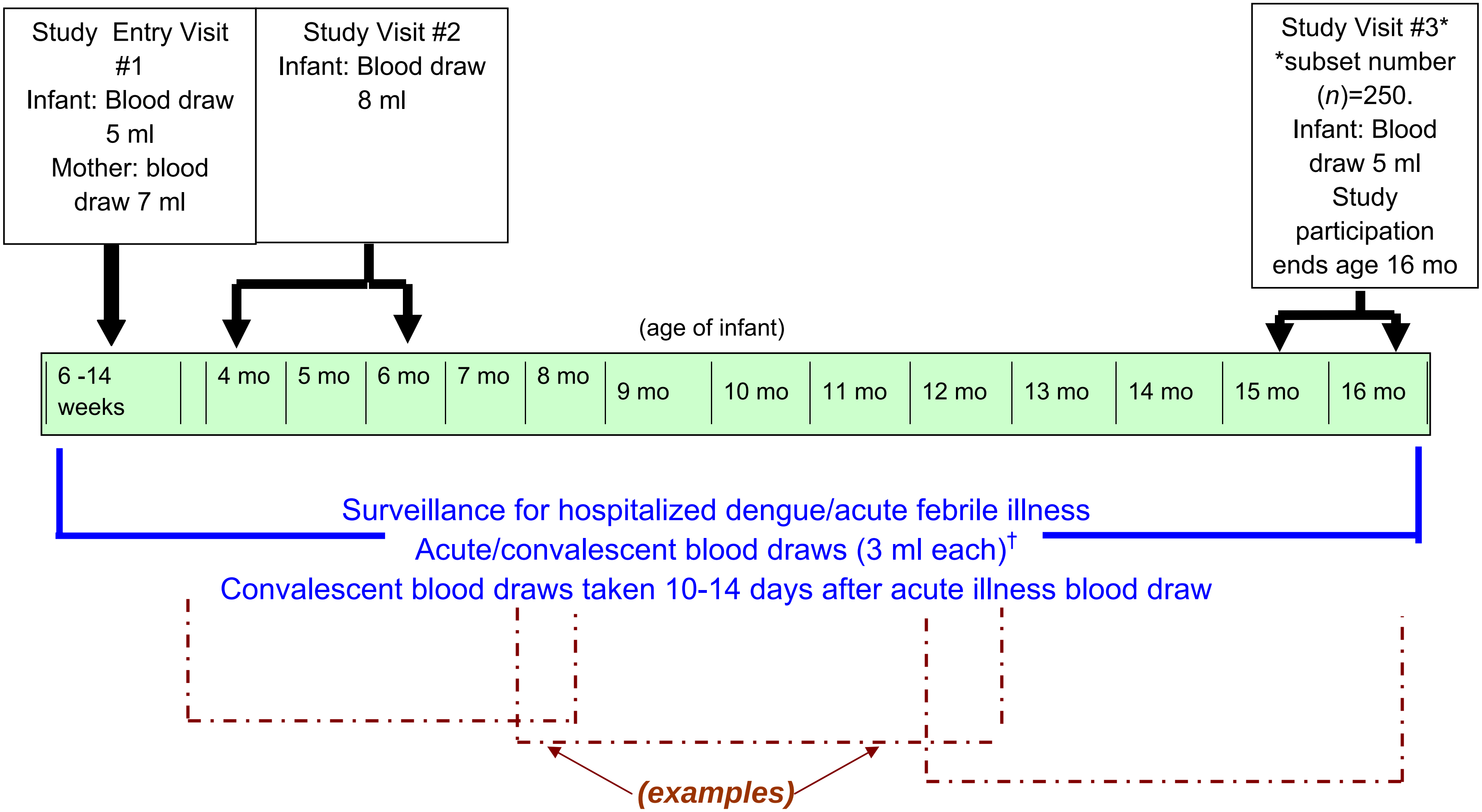

Additional surveillance for non-hospitalized dengue/acute febrile illness for 5 months between June-October of a calendar year. Acute/convalescent blood draws (3 ml each)†. Convalescent blood draws taken 10-14 days after acute illness blood draw.† Participating infants cannot have > 2 paired (acute and convalescent) surveillance blood samples taken while in the study.

1 KEY ROLES

Principal Investigator: (Site investigator)

Maria Rosario Z. Capeding, M.D., Head Department of Microbiology, Consultant Pediatrics and Infectious Disease Clinical Department,

Research Institute for Tropical Medicine (RITM), Alabang, Muntilupa City, Philippines
Tel/Fax: (632) 7724916
Email: rcapeding@ritm.gov.ph or lerose@info.com.ph

Institutions:

Institution: Research Institute for Tropical Medicine

Address: Alabang, Muntilupa City, Philippines

Contact Person: Maria Rosario Z. Capeding, M.D.

Tel/Fax: (632) 7724916
E-mail: rcapeding@ritm.gov.ph or lerose@info.com.ph

Institution: Center for Infectious Disease and Vaccine Research (CIDVR), University of Massachusetts Medical School (UMMS)

Address: Rm S5-326, 55 Lake Ave. North, Worcester, MA, USA 01655

Contact Person: Daniel H. Libraty, M.D.

Tel: 508-856-4182

Fax: 508-856-4890

E-mail: daniel.libraty@umassmed.edu

Institution: San Pablo City Health Office

Address: San Pablo City, Laguna, Philippines

Contact Person: Job Brion, M.D.

Tel: (632) 049- 5628116

Fax: (632) 049-5627874

E-mail: jdbrion@laguna.net or jdbrion@yahoo.com

Institution: Department of Virology, Armed Forces Research Institute of Medical Sciences (AFRIMS)

Address: 315/6 Rajvithi Rd., Bangkok 10400, Thailand

Contact Person: LTC Mammen P. Mammen, M.D.

Tel: 66-2-644-5644

Fax: 66-2-644-4760

E-mail: mammenmp@afrims.org

Optional:

2 BACKGROUND INFORMATION AND SCIENTIFIC RATIONALE

2.1 Background Information

In dengue endemic countries such as the Philippines, there is an urgent need for a dengue vaccine; however, there is also major concern that widespread use of a vaccine may increase the risk for dengue hemorrhagic fever (DHF). Thus, it is critical that reproducible correlates of protective immunity be identified. The goal of this proposal is to identify correlates of protective immunity against dengue in a prospective study of DV infections during infancy. Unlike older children, infants manifest symptomatic dengue (including DHF) following primary DV infections that are modified by pre existing, maternally-acquired, humoral immunity. The unique immunological conditions of infancy provide the most unadulterated setting to define protective immune correlates. **We hypothesize that specific levels of maternally-acquired neutralizing antibodies (Abs) are associated with protection against symptomatic dengue during infancy, and protective Ab levels differ among the DV serotypes. When maternally-derived anti-DV Abs fall below protective levels, we hypothesize that virus replication following a primary DV infection can stimulate a convergence of host pathogenic factors unique to infancy and lead to DHF.**

Dengue is an emerging arboviral disease caused by infection with one of the dengue viruses (DVs), a group of four antigenically related mosquito-borne flaviviruses. The DVs are widespread throughout the tropics and produce significant morbidity and mortality in at least 1/2 million persons/year [1]. The World Health Organization (WHO) has categorized dengue as a high priority emerging viral disease, as over 1 billion people in the world are at risk for DHF. Infection with any 1 of the 4 DV serotypes (DV-1, DV-2, DV-3, DV-4) can produce a spectrum of illness. The manifestations of a DV infection range from asymptomatic or mild febrile illness, to the more symptomatic dengue fever (DF), to the most severe and potentially life-threatening illness, DHF. The cardinal feature of DHF, as in other viral hemorrhagic fevers, is rapid and severe vascular dysregulation. The vascular dysfunction contributes to thrombocytopenia, a coagulopathy, and, most importantly, a transient increase in vascular permeability. The circulating blood volume is reduced, hemoconcentration occurs, and, in severe cases, hypovolemic shock develops [2, 3]. Fluid resuscitation and intensive care can be life saving, but specific treatments are not available and often access to appropriate care is limited. So far, the main preventive strategy in dengue endemic countries is mosquito control of the primary DV vector, *Aedes aegypti*. Despite some success in a few countries, widespread mosquito control efforts have been difficult to sustain. Development of a vaccine is seen as the only practical strategy for effective prevention and long-term control of dengue disease. The vaccines under development that are likely to be considered for use in dengue endemic countries are live-attenuated or chimeric viruses combined in a tetravalent formulation. Beyond the problems of interference in multivalent viral vaccines, there are additional and somewhat unique

immunological issues in dengue that will pose challenges to mass, public health, vaccination campaigns against dengue.

A primary infection with one DV serotype typically produces long-term protective immunity against the homologous serotype. After a short period of cross-protection, individuals who have recovered from a primary DV infection are fully susceptible to infection and disease by heterologous serotypes (secondary infection) [4, 5]. In fact, the relative risk of developing DHF is enhanced 5 to 500-fold following sequential, heterotypic DV infections [6-8]. For dengue vaccines, even tetravalent ones, the key unanswered question remains what are the vaccine-induced immune responses that will recapitulate natural infection induced protective immunity and avoid enhancement of disease severity? The potential pitfalls of a dengue vaccination campaign are that ineffective or short-lived vaccine induced immune responses would not only fail to protect against disease, but could enhance disease severity in vaccinated individuals. Given these consequences, it is imperative that clear and reproducible correlates of protective immunity are well defined before large scale dengue vaccine clinical trials and public health vaccination programs begin.

The majority of clinical studies on dengue have focused on secondary DV infections and identifying risk factors for development of DHF. There have been 5 community-based, prospective, studies of dengue reported in the literature [6, 9-12]. All have consistently found that pre existing anti DV immunity, generally reflecting prior infection with heterologous DV serotypes, is associated with development of DHF. In a recent paper [13], we focused on examining correlates of protective anti DV immunity in a prospective cohort study of schoolchildren in Thailand. We found that the presence of pre existing neutralizing Abs directed against the infecting DV serotype (either a prototype strain or the infecting virus isolate) was not a good correlate of disease protection for secondary D1V, D2V, and D3V infections. So far, the protective capability of dengue vaccine candidates has been inferred by their ability to induce detectable neutralizing Ab responses. Our data suggest that the presence of neutralizing Abs is unlikely to be an adequate immune correlate for selecting, testing, and using dengue vaccines. Vaccines inducing and sustaining higher levels of neutralizing Abs would be considered better than those inducing lower Ab levels. What levels of neutralizing Ab to a given DV serotype can be considered “protective” or “adequate” is the primary unanswered question that this proposal aims to address.

For many different viruses, animal models, and challenge routes, passive Ab transfer experiments have consistently shown a good correlation between in vivo protection and the Ab neutralizing activity measured in vitro [14]. Even though the correlation does not necessarily imply that the mechanism of neutralization in vitro is the only, or even predominant, mechanism of protective activity in vivo. As there is no animal model for DHF, studies of dengue in infants born to DV immune mothers are the best approximations of passive immunization experiments. A careful examination of dengue in infants is the most unadulterated way to identify protective immune correlates in a clinical study, and cannot be replicated in studies of secondary DV infections. There have been very few studies of infant dengue to date, and none focusing on correlates of protective immunity.

Insight into protective immunity in dengue can also be gained by studying the converse process, immune enhancement of disease severity. DHF among infants (age < 12 months) was

first reported in 1970 [8]. In contrast to childhood DHF, DHF among infants occurs almost exclusively in the setting of a primary DV infection. Epidemiological data from four Southeast Asian countries have shown a similar age distribution pattern for hospitalized infant DHF [15]. There are few cases at < 3 months and the largest number of cases is typically between 6-9 months. The number of infant DHF cases then decreases variably over the remainder of the first year of life. A unifying explanation of childhood DHF following secondary DV infection and infant DHF following primary DV infection has been the presence of circulating anti-DV Abs, either actively acquired (from earlier primary infection) or passively acquired (from maternal-fetal transfer). When DV serotype cross reactive Abs are at sub neutralizing concentrations, it is postulated that they facilitate DV entry into Fc-receptor-bearing cells, thereby increasing the virus burden and severity of disease. This has been termed Ab dependent enhancement (ADE) of infection. ADE of DV infection in monocytes and other Fc receptor bearing cells has been demonstrated in some in vitro and ex vivo studies [16, 17]. ADE of DV infections has been considered the only risk factor that infants have for developing DHF, and therefore the only unifying explanation for immune enhancement of dengue disease severity. However, there has been only one small infant study using ex vivo blood samples in an attempt to explain the epidemiological findings [18]. A careful look at the limited data available on infants to date reveals many unanswered questions.

We feel that a carefully designed, comprehensive, prospective study of DV infections over the first 16 months of life will successfully avoid some of the pitfalls of previous studies, and narrow the gaps in knowledge regarding immune correlates of protection against symptomatic dengue and delineation of important factors in the pathogenesis of DHF. To that end, a collaborative and international consortium has been formed to advance dengue research in the Philippines. The research will generate vital data for the effective testing of dengue vaccines and future public health vaccination campaigns throughout the region.

2.2 Scientific Rationale

Our long-term aim is the prevention of DHF and hospitalized dengue in children with a safe and effective vaccine. This collaborative study will provide immediately relevant, sound, scientific data useful for selecting and testing dengue vaccine candidates in Phase III clinical trials, as well as for future implementation of dengue vaccine campaigns. A prospective study of primary DV infections during infancy provides a unique opportunity to identify correlates of immune protection or immune enhancement not provided by studies of secondary DV infections. While some vaccines have been developed without identifying correlates of immune protection, the potential harm caused by ineffective or incomplete immunity to DVs makes a better understanding of immunity in dengue critical for the advancement of vaccination campaigns. Results from this study are likely to have a significant impact on future dengue vaccination testing and administration policies in the Philippines and other Southeast Asian countries.

There is no animal model for DHF or DF. As such, prospective human studies of natural DV infections are critical for advancing dengue vaccine development and usage.

2.3 Potential Risks and Benefits

2.3.1 Potential Risks

a) Phlebotomy –

- Immediate risks- can infrequently cause temporary bruising, erythema, pain, infection, or bleeding.
- Long range risks- none.
- Rationale- There are no direct health benefits from the collection of blood samples to the subjects. The minimal risks of phlebotomy are reasonable and necessary to successfully address the study objectives, namely identify immunological correlates of protection to dengue.
- To diminish the risk of phlebotomy, only adequately trained study personnel will draw blood. Blood volumes taken from the study subjects fall within the Office of Human Research Protections guidelines for minimal risk studies [19]. Alternative procedures are not feasible.

2.3.2 Known Potential Benefits

i) Participation in study may improve outcome in DV infections.

The morbidity and mortality from DHF is often from a delay in seeking medical care. Parents in the study will be educated and encouraged to bring their infants to medical attention for febrile illnesses. Early medical evaluation of dengue can offset the severe consequences of the disease.

ii) Study will provide rapid dengue diagnostic testing for acute febrile illnesses.

In infants with an acute febrile illness identified either during hospitalization or during outpatient surveillance during the peak DV transmission season, an acute illness blood sample will be obtained and tested using a DV IgM enzyme-linked immunosorbent assay (EIA) and DV reverse transcriptase-polymerase chain reaction (RT-PCR). The results of the assays and an interpretation of their significance will be reported to the parent/guardian and healthcare provider of the ill child within 2-3 days. These diagnostic tests will be performed at no cost to the subject. If the child requires additional medical attention, this information will be provided to the appropriate healthcare provider to assist in the clinical management.

iii) Compensation at study visits-

a) Study Visit 1- study will pay for transportation to and from City Health Office and provide an infant digital thermometer and multivitamin with iron for mothers.

b) Study Visit 2- study will pay for transportation to and from City Health Office and provide a food tray with utensils, sipping cup, and educational information on dengue.

c) Study Visit 3 (if selected)- study will pay for transportation to and from City Health Office and parents will receive a framed photograph of their child.

d) Study exit (age 16 months)- all children will receive a toy, not valued greater than $1 USD.

3 OBJECTIVES

Primary Objective

- The first aim of this project is to define levels of serotype-specific neutralizing Abs associated with protective immunity against symptomatic dengue. Neutralizing Ab titers in blood samples collected from Filipino infants before DV infection, and predicted neutralizing Ab titers at the time of illness, will be correlated with disease severity rankings and peak viremia levels. Neutralizing Ab titers at which infants developed symptomatic dengue will be determined.

Secondary Objectives

- The second aim is to delineate risk factors contributing to the pathogenesis of DHF in infants. From pre-illness blood samples, the association of Ab neutralizing capacity or enhancement of infection with DHF will be measured while controlling for other potential covariates. The covariates will include measures representing viral load and infant host immune responses.
- The third aim is to strengthen the capacity for diagnosis and research on dengue in the Philippines. A collaborative and international consortium has been formed to advance dengue research in the Philippines. The research will generate vital data for the effective testing of dengue vaccines and future public health vaccination campaigns throughout the region.

4 STUDY DESIGN

This is a prospective natural history study to define the levels of maternally-acquired neutralizing Ab and other correlates of immunity that may be protective against the development of dengue hemorrhagic fever in infants. Approximately 10,000 mother-infant pairs will be enrolled to achieve a continuous study cohort of approximately 4,000 children less than 16 months of age. Children leave the study when they reach 16 months of age and are replaced by new participants at 6-14 weeks of age.

I. Recruitment

We will recruit study participants among 3rd trimester pregnant women receiving pre-natal care and women with newborns receiving post-natal care in San Pablo City. Educational and informational sessions will also be organized for midwives and other healthcare delivery personnel in San Pablo City. Through midwives, local healthcare providers, and the network of public health clinics, study advertisement and informational materials will be distributed to these women (Appendices C and D). Investigators will also hold community meetings several times per year for expectant mothers and parents of infants less than 14 weeks to discuss the study goals and potential risks and benefits. Details of study participation will be presented individually to each interested mother. The study protocol and consent forms in Tagalog will be reviewed in detail, questions will be addressed, and written informed consent will be obtained at study visit #1. Up until infant age of 14 weeks, there will be no pre-specified time limit for mothers to decide whether they would like to participate. The consent form in Tagalog will be signed by the mother, and witnessed by study personnel and another person. Participants may leave the study at any time without penalty.

II. Scheduled visits

i) Study Visit #1/Study entry- Mothers and infants will enter the study when the infant is between the ages of 6-14 weeks*. We anticipate that the majority of infants will enter the study at ages 6, 10, or 14 weeks, when they are scheduled for one of their Extended Programme in Immunization (EPI) vaccinations (diphtheria/pertussis/tetanus, polio). After informed consent is obtained, data on the mother and the birth will be collected. In all infants, an anticoagulated blood sample will be collected upon study entry for plasma and peripheral blood mononuclear cells (PBMC) isolation. A serum sample from the mother will be collected at the same time or within 2 weeks of the infant blood sample.
(* Except on study initiation, when over the first 5 months of the study, infants between the ages of 6-24 weeks will be allowed to enter the study. This is to achieve an adequate sized study cohort for surveillance during the first dengue season).

Data on mothers to be collected

Form data	Date, Study ID no, subject initials, Study Visit #, Location code
Demographics	Name, address, telephone, age, date of birth
Past medical hx	Pre-eclampsia/eclampsia, gestational diabetes, tuberculosis (active), dengue (age of occurrence)
Current medical conditions	Diabetes, asthma, lupus/autoimmune ds, tuberculosis (active), medications
Birth/delivery	gravida/para, normal vaginal delivery, complicated vaginal delivery, or C-section, transfusion at delivery
Feeding	Breastfeeding alone, breastfeeding+formula, formula alone; if breastfeeding, quantify times/24 h period

Data on infants to be collected

Form data	Date, Study ID no, subject initials, Study Visit #
Demographics	Name, age, date of birth, gender
Past med hx	Hospitalization between birth and current visit, reason, and location?<br>Febrile illnesses lasting >3 days between birth and current visit?
Medications	Current medications
Measurements	Height, weight, temperature
Vaccination hx	Vaccinations received to date from EPI card

ii) Study Visit #2- Study Visit #2 will take place when the infant is between the ages of 4-6 months. At this time, data on infant health will be obtained. In all infants, an anticoagulated blood sample will be collected for plasma and PBMC isolation.

Data on infants to be collected

Form data	Date, Study ID no, subject initials, Study Visit #
Demographics	Date of birth, gender
Past med hx	Hospitalization since last study visit, reason, and location?<br>Febrile illnesses lasting >3 days since last study visit?
Medications	Current medications
Measurements	Height, weight, temperature
Vaccination hx	Vaccinations since last study visit from EPI card
Feeding	Breastfeeding alone, breastfeeding+formula/solids, formula/solids alone; if breastfeeding, quantify times/24 h period.

iii) Study Visit #3- At age 15-16 months, 250 infants/year will be selected to return for a 3rd study visit. The 250 infants will be randomly selected from those without prior symptomatic dengue and whose study visits 2 and 3 span a significant portion of the peak DV transmission season (June-October). Data on infant health will be obtained and an anticoagulated blood sample for plasma will be collected.

Data on infants to be collected

Form data	Date, Study ID no, subject initials, Study Visit #
Demographics	Date of birth, gender
Past med hx	Hospitalization since last study visit, reason, and location?<br>Febrile illnesses lasting >3 days since last study visit?
Medications	Current medications
Measurements	Height, weight, temperature
Vaccination hx	Vaccinations since last study visit from EPI card
Feeding	Breastfeeding alone, breastfeeding+formula/solids, formula/solids alone; if breastfeeding, quantify times/24 h period.

III. Surveillance

i) Hospitalized dengue- Surveillance for DV infections that result in hospitalization will occur year round and throughout the entire period of an infant's study participation (ages 6 weeks-16 months). Upon study enrollment, parents will be given a study identification card that will have their infant's unique identification number. When any infant in the study presents to one of the 7 hospitals serving San Pablo City, they will be identified as a study participant and their identification number and date/time of presentation will be transmitted to the study coordinating center at the City Health Office. Study personnel will then screen infants in the study who were admitted for acute febrile illnesses. Those infants will have history and clinical data abstracted, and an acute illness blood sample (serum) taken for dengue diagnostic testing. A convalescent blood sample (serum) will also be taken 10-14 days later.

Data to be collected on infants hospitalized with acute febrile illnesses

Form data	Date, Study ID no, subject initials, location code
Demographics	Date of birth, gender
Illness type	Fever onset, symptoms, signs
Hospital course	Clinical, laboratory, and radiographic data abstracted from medical record at end of hospitalization

ii) Non-hospitalized dengue- Each year during the peak DV transmission season from June to October, we will also conduct surveillance for DV infections that do not result in hospitalizations. During this time period, parents will be encouraged to bring their infants to the barangay health stations, local doctor's offices, or the City Health office if they develop an acute febrile illness. We will rely primarily on parents bringing their infants to the City Health office for febrile illnesses during the peak dengue season. In addition, nearly all pediatric health care providers in San Pablo will be aware of the study through education and information distributed through the local medical society and hospitals. Parents will be

reminded to present their study identification cards if they bring their infant with a fever > 38.0°C to a barangay health station or a private doctor's office. The health care personnel will be instructed to call a central telephone number where study personnel will coordinate transportation to the City Health office. There, study personnel will assess the infants, complete a questionnaire, and obtain an acute illness blood specimen (serum) for dengue diagnostic testing in addition to any routine clinical tests that may be requested by the treating nurse or physician. If the acute illness serum sample returns positive by DV IgM or RT-PCR, study personnel will arrange for collection of a convalescent blood specimen (serum) 10-14 days later.

IV. Outcomes

i) Primary outcome- Identification of DV infection and classification of disease severity.

Study subjects will be classified into the clinical categories shown in the table on the next page. Clinical categories I and II will be identified by surveillance for hospitalized dengue. Category III will be identified by surveillance for non-hospitalized dengue during the peak DV transmission season. Category IV will be identified by hemagluttination inhibition (HAI) seroconversion between study visits #2 and #3 in the 250 infants selected for blood sample collection at study visit #3.

ii) Secondary outcome- estimation of peak viremia levels.

We have previously found that viremia levels obtained within 72 hours of symptom onset in dengue are a good approximation of peak viremia levels [20-22]. A serotype specific, fluorogenic, quantitative RT-PCR assay will be performed on the acute illness serum samples collected within 72 hours of symptom onset from infants in Categories I-III.

Clinical Category	Diagnostic Criteria	Lab Diagnosis of DV infection	Serotype of infection	Primary DV infection
I. Hospitalized DHF	1. Acute febrile illness* requiring hospitalization, and 2. Lab diagnosis of DV infection, and 3. Meets WHO criteria for DHF.	1. + DV RT-PCR from acute illness serum sample, or 2. + DV IgM from acute illness serum sample.	Determined from RT-PCR	IgM/IgG EIA pattern in paired acute and convalescent samples
II. Hospitalized DF	1. Acute febrile illness* requiring hospitalization, and 2. Lab diagnosis of DV infection, and 3. Does not meet WHO criteria for DHF.	1. + DV RT-PCR from acute illness serum sample, or 2. + DV IgM from acute illness serum sample.	Determined from RT-PCR	IgM/IgG EIA pattern in paired acute and convalescent samples
III. Non-hospitalized DF	1. Acute febrile illness* brought to medical attention, and 2. No hospitalization, and 3. Lab diagnosis of DV infection.	1. + DV RT-PCR from acute illness serum sample, or 2. + DV IgM from acute illness serum sample.	Determined from RT-PCR	IgM/IgG EIA pattern in paired acute and convalescent samples
IV.Mild/asymptomatic DV infection (subset analysis)	1. No symptomatic febrile illness during surveillance period identified as a DV infection, and 2. DV seroconversion over surveillance period.	1. > 4-fold rise in anti-DV HAI Ab titers over scheduled blood draws encompassing the surveillance period.	Infer from neut Ab patterns to DVs 1-4	Infer by age
V. No evidence of DV infection	none of the above			

* Temperature ≥ 38.0°C.

5 Study Population

5.1 Selection of the Study Population

Up to 10,000 healthy mother-infant pairs will be enrolled over a 4 year period. Infants will be between 6-14 weeks of age at enrollment and will be followed until they are 16 months of age. The goal is to maintain a continuous cohort of approximately 4,000 infants at any time, replacing those who go off study at 16 months of age with new subjects. Thus, study subjects will be infants, male or female, between the ages of 6 weeks to 16 months, of any race or ethnicity. The infants' mothers will also be included as study subjects (no age restriction). Infants with congenital medical conditions or born to HIV seropositive mothers (obtained by history) will be excluded so that these factors do not become confounding variables in the study. Pregnancy HIV testing will not be required. The study population will be drawn from infants born to mothers residing in the San Pablo City Health District, Laguna, Philippines. Study subjects will be recruited and enrolled in outpatient public health clinic settings.

5.2 Inclusion/Exclusion Criteria

Inclusion Criteria for infants:
a) Infants born to mother residing in San Pablo Health District.
b) Planned residence in San Pablo Health District for at least 1 year.
c) Age 6-14 weeks*.
d) Informed consent.
e) General good health

* Except on study initiation, when over the first 5 months of Year 1 (est. Feb-June 2006), infants between the ages of 6-24 weeks will be allowed to enter the study).

Exclusion Criteria for infants:
a) Infant born with congenital medical disorder.
b) Mother known to be HIV seropositive.

Mothers of infants meeting the inclusion/exclusion criteria will also be enrolled, and this will include breastfeeding women. There are no requirements regarding contraception methods for the enrolled mothers.

6 STUDY PROCEDURES/EVALUATIONS

6.1 Study Procedures

Subjects will receive a study ID code identifying the sequence of enrollment. The alphanumeric study ID code will be generated automatically on the study database. A database linking identifying information with the study ID code will be maintained under restricted access, secure conditions, at RITM. Parents will receive a study ID card listing the infant's name, date of birth, and study ID code for use at future scheduled and surveillance study visits. Blood samples collected at scheduled or surveillance study visits will be identified by a different alphanumeric specimen ID code. Data will be recorded on paper case report forms (CRFs) appropriate to the study or surveillance visit. Blood sample collection data and diagnostic test results will also be recorded on paper CRFs. The data collection system will be compliant with all applicable regulations.

Time of participation

The expected total duration of an infants' participation is between 50-58 weeks, from the age of 6-14 weeks to 16 months. The total duration of the mothers' participation is one study visit upon study entry.

Study visit	Time required
Study Visit #1 (infant)	1.5 hours
Study Visit #1 (mother)	1.5 hours (concurrent with infant)
Study Visit #2 (infant)	1 hour
Study Visit #3 (infant)	1 hour

Data management

The paper CRFs will be kept in locked cabinets at the central field office, and first translated into electronic format (eCRFs) at the field office. The paper CRFs will then be transported to RITM for second data entry into eCRFs. This double data entry will serve as an initial quality assurance (QA) assessment. The need for continuation of double data entry will be re-assessed after 6 months. The eCRFs will be linked to the study database.

Analytic files will retain only the unique study ID code attached to each subject in the study. Data collection will follow a set of standard operating procedures that include field and office editing, and operation checks to ensure the validity of the data and adherence to the protocol. One electronic file will be created in MS Access for each CRF (including CRFs for diagnostic test results). CRFs will be re-abstracted at a rate of 1 in 20 in the field. Discordance will be noted and any apparent systemic errors at the field level will be investigated for possible needs to retrain staff. Data from the field will be submitted to the Data Management Unit at RITM for double encoding as noted earlier. After this initial check, a validation program

(developed by the programmer) will be run to check for inconsistencies in data. Initial tables are generated for final checking of data from CRFs before merging and analyzing databases.

The study database will be stored and maintained by the Data Management Unit at RITM. A back-up copy of validated data will be stored on CDs in a secure location at an external site. Back-up copies of validated data will also be sent to UMMS for secure storage. Data will be stored under secure conditions for a minimum of 10 years after completion of the study.

6.2 Laboratory Evaluations

6.2.1 Laboratory Evaluations/Assays

Scheduled Visits

Time point	Blood collection volume	Aliquots/Assays
Study Visit #1 (infant)	5 ml (anticoagulated tube)	Plasma: 1.0 ml neut Ab assay, 1.0 ml ADE assay, 2.5 ml for repeat assays/storage (total 4.5 ml)<br>PBMC: $5x10^6$ cells for activation and cytokine assays.
Study Visit #1 (mother)	7 ml (serum separator tube)	Serum: 1.0 ml neut Ab assay, 2.0 ml for repeat assays/storage (total 3.0 ml)
Study Visit #2 (infant)	8 ml (anticoagulated tube)	Plasma: 1.0 ml neut Ab assay, 1.0 ml ADE assay, 0.5 ml HAI assay, 2.0 ml for repeat assays/storage (total 4.5 ml)<br>PBMC: $8x10^6$ cells for activation and cytokine assays.
Study Visit #3 (infant)	5 ml (anticoagulated tube)	Plasma: 0.5 ml HAI assay, 1.0 ml neut Ab assay, 2.5 ml for repeat assays/storage (total 4.0 ml)

Surveillance (hospitalized or non-hospitalized dengue surveillance)*

Time point	Blood collection volume	Aliquots/Assays
Acute illness sample	3 ml (serum separator tube)	Serum: 0.25 ml IgM EIA, 0.25 ml RT-PCR, 1.0 ml for repeat assays/future RT-PCR and IgM/IgG EIA/storage
Convalescent sample (10-14 days later)	3 ml (serum separator tube)	Serum: 0.5 ml for IgM/IgG EIA, 1.0 ml for repeat assays/storage

* Participating infants cannot have > 2 paired (acute and convalescent) surveillance blood samples taken while in the study.

The proposed amount of blood drawn from study participants poses no more than minimal risk as defined by Office of Human Research Protections guidelines [19]. The amount of blood drawn does not exceed the lesser of 50 ml or 3 ml/kg in an 8 week period and collection does not occur more frequently than 2 times/week.

Assay descriptions:

Virus RT-PCR- A qualitative, serotype specific, DV RT-PCR assay will be performed on all acute illness serum samples. The assay technique has been previously published [23]. Results will be available within 3 days, and outpatient subjects with a positive DV RT-PCR will have a convalescent blood sample collected. Paired acute and convalescent serology will be performed to confirm primary DV infections (see below).

Serology- An anti-DV IgM EIA will be done on all acute illness serum samples collected. The results will be available within 3 days, and outpatient subjects with a positive DV IgM will have a convalescent blood sample collected. Paired acute and convalescent sera will be tested for anti-DV IgM/IgG by EIA using standard techniques and criteria to confirm primary DV infections [24].

At the conclusion of the year, plasma samples from the 250 subjects with 3 scheduled study visits will be identified. These subjects will not have had an earlier recognized symptomatic DV infection. The plasma samples that span a significant part of the peak DV transmission season will be tested for anti-DV Abs by HAI serology [25] to identify those with clinically unrecognized seroconversions.

Neutralizing Ab assays- All subjects in the clinical study with evidence of DV infection (Categories I-IV) will have neutralizing anti-DV Ab titers determined in their pre-infection blood draw. The neutralizing Ab titers will be determined using a standard plaque reduction assay to reference DV isolates serotypes 1-4 [13]. We have previously shown that neutralizing Ab titers to reference DV isolates correlate well with neutralizing Ab titers to the actual infecting virus isolate [13]. The 50% plaque reduction neutralization titer will be calculated using 4-parameter curve fitting analysis.

Peak viremia determinations- We have found that viremia levels obtained within 72 hours of symptom onset in dengue are a good approximation of peak viremia levels [20-22]. A serotype specific, fluorogenic, quantitative RT-PCR assay will be performed on the acute illness serum samples collected within 72 hours of symptom onset from infants in Categories I-III.

Ab-dependent enhancement of infection assay (ADE)- All subjects in the clinical study with evidence of DV infection or likely exposure (Categories I-IV) will have an ADE Ab assay performed using their pre-infection blood draw.

PBMC activation- We recognize that limited numbers of PBMC will be available for study from the infant blood draws. Therefore, we plan to use modern multiplex assay techniques to get an overview of DV-induced immune activation patterns in infant PBMC. We will first perform the assays on serial collected PBMC (study visits 1 and 2) from 30 subjects without evidence of DV infection (Category V). This will allow us to refine the assays. Once optimized, the assays will be done on the pre-infection PBMC from all subjects with evidence of DV infection (Categories I-IV).

PBMC will be stimulated in vitro with DV and controls. At the early and late time points, cell culture supernatants will be collected and cellular RNA will be isolated. Pro- and anti-inflammatory cytokine/chemokine levels will be determined in the supernatants using a multiplex bead assay. 9 cytokines/chemokines will be assayed in a single sample using the preconfigured panel shown below:

Category	Mediators
Pro-inflammatory mediators	IL-1β, IL-6, TNF-α, IFN-γ, IL-12 p70
Anti-inflammatory mediators	IL-4, IL-10, IL-13
CC chemokines	MCP-1

Regulation of vascular permeability is an important factor in DHF pathogenesis, and modulation of anti-viral and antigen-presentation functions are well established paradigms in many virus-host interactions. We will use cellular RNA to investigate these additional immune response gene expression profiles by quantitative, real-time, fluorogenic RT-PCR. Gene expression levels will be compared to established housekeeping genes. The genes to be investigated are shown below:

Category	Gene profiles
Vascular permeability mediators	TXAS, HIF-1α and VHL, VEGF
Anti-viral activities	OAS2, OASL, MX1, ISG15
MHC Class I antigen processing	PSMA2, β2M, TAPBP
MHC Class II antigen processing	CIITA, Ii

The strategy outlined above will allow us to investigate different relevant aspects of the infant response to DV infection with limited sample volumes and cell numbers.

IFN-γ enzyme-lined immunoSPOT- Although pre-existing DV T-cell reactivity is not expected in infants with primary DV infections, potential T-cell responses to DV will be examined as part of the comprehensive assessment of DHF risk factors. There is limited literature suggesting that maternal T-lymphocytes may be transferred to infants by breastfeeding [26, 27]. We will first perform the assay on serial collected PBMC (study visits 1 and 2) from 20 breastfed infants without evidence of DV infection (Category V) and born to DV-seropositive mothers. This will allow us to see if indeed DV-reactive T-cells are detectable, and, if so, investigate possible age-related changes. Then, the assay will be done on the remaining 2-3x10^6 pre-infection PBMC from the subjects with evidence of DV infection (Categories I-IV).

6.2.2 Specimen Collection, Preparation, Handling and Shipping

6.2.2.1 Instructions for Specimen Preparation, Handling, and Storage

Bar code labels with log numbers will be prepared in advance for the scheduled blood draws and acute/convalescent illness blood draws. Upon enrollment, mother and infant will each be given a unique study identifier. When blood is drawn from a study subject, one bar code label is automatically entered or placed in the data record and a duplicate bar code label is placed on the specimen. The bar code log numbers will be scanned into the specimen receiving database, which contains the study identifiers. Aliquots of plasma samples will be stored in -70°C freezers, and PBMC will be stored in liquid nitrogen at -120°C. Blood specimens will be stored at RITM, or at UMMS following RITM permission. Given the large number of blood specimens over the 4-year study period, it will be impossible to store all samples over the duration of the study and analysis periods. Samples from subjects without DV infections, and not identified as potential matched controls, will be destroyed following appropriate biosafety precautions on a yearly basis.

6.2.2.2 Specimen Shipment

Specimens for QA checks of dengue diagnostic assays (EIA, RT-PCR) will be sent from RITM to AFRIMS, Department of Virology on a bimonthly basis over the first 2 years of the study. Specimens for QA checks of HAI serology and neutralizing Ab assays will be sent from RITM to AFRIMS, Department of Virology on an annual basis. Specimens for ADE assay, and PBMC/T-cell assays will be sent from RITM to CIDVR, UMMS on a semi-annual basis. All specimens will be coded, anonymous, samples, and sent on dry ice. Shipment protocols will follow the appropriate International Association Transportation Authority packing and shipping regulations.

7 STATISTICAL CONSIDERATIONS

7.1 Study Outcome Measures

- In order to achieve the first and second objectives of the study (Section 3), the primary outcome measure will be identification of DV infections and classification of dengue disease severity (described in Section 4, subpart IV). The primary outcome measure is categorical. Subjects with hospitalized DHF (Category I) will be rated as severity=5, and each step downward in severity category will be rated by a decrease in one point on a severity scale (4, 3, etc.).

- The secondary outcome measure, peak viremia level with DV infection, is also linked to the first and second objectives. It is a continuous variable associated with disease severity, the primary outcome measure.

Study Variables

Variable	Measurement	Time obtained
Neutralizing Ab titers	Continuous and categorical	Study visits 1 and 2 (pre-illness)
ADE Ab assay	Continuous and categorical	Study visits 1 and 2 (pre-illness)
PBMC activation assays	Continuous and categorical	Study visit 1 or 2 (use single time point before illness for analysis)
IFN-γ enzyme-linked immunoSPOT assay	Continuous and categorical	Study visit 1 or 2 (use single time point before illness for analysis)
Epidemiological and clinical data collected on case report forms	Binary and categorical	Study visits 1 and 2, and at time of illness

7.2 Sample Size Considerations

- Dengue surveillance in San Pablo City over the past 4 years has demonstrated annual incidence rates of infant hospitalized dengue between 0.05-0.25%. The

population of infants between 0-16 months of age in San Pablo City is approximately 16,000-20,000. A study cohort continuously maintained at 4,000 subjects in this age range will capture infant hospitalized dengue rates of 0.1-0.2% with 90% confidence (EpiInfo, www.cdc.gov/epiinfo).

- To identify subjects with mild/asymptomatic dengue, we will measure asymptomatic seroconversion in a subset of 250 subjects/year. We anticipate a 1-3% yearly incidence of mild/asymptomatic dengue [12]. A sample of 250 subjects will capture a mild/asymptomatic dengue incidence rate of 1-3% with 90-95% confidence (EpiInfo, www.cdc.gov/epiinfo).
- Based on these sample sizes and assumptions, we anticipate capturing the following number of subjects over a 4-year period:

Hosp DHF	Hosp DF	Non-hosp DF	Mild/asymptomatic seroconversion (from random sample of *n*=250/year)
11	11	44	32

At the end of the study period, we expect to have nearly *n*=100 infants with DV infections, and a much larger pool without evidence of DV infections to select matched controls (*n*=200 or 300). Data analysis with these anticipated numbers is detailed in section 7.4.

7.3 Participant Enrollment and Follow-Up

After start-up, enrollment and subject accrual will occur on a continuous, rolling, basis in order to maintain the cohort goal of 4,000 infants between the ages of 6 weeks to 16 months. New infants between the ages of 6-14 weeks will be enrolled to replace those disenrolled after reaching age 16 months. There is no additional follow-up for subjects after leaving the study at age 16 months.

7.4 Analysis Plan

I. *First study objective-* correlate pre-existing, maternally-derived, neutralizing Ab titers against the infecting DV serotype with disease severity.

Subjects with hospitalized DHF will be rated as severity=5, and each step downward in severity category will be rated by a decrease in one point on a severity scale (4, 3, etc.). Neutralizing Ab titers determined in the pre-infection blood draws, and predicted neutralizing Ab titers at the time of illness based on a kinetic model of anti-DV Ab clearance (see below), will be used for analysis. The association between pre-existing neutralizing Ab titers (raw and log-transformed) and severity of DV infections will be evaluated by fitting partial proportional odds multivariate longitudinal models [28, 29]. When needed, we will also use generalized estimating equations [30, 31], clustering on patient observations over time, while using a logit link function and unstructured error correlation structure. Analyses will be performed separately

for each DV serotype, as well as with all serotypes pooled (and serotype included as a candidate term in the model).

We will also compare pre-existing, maternally-derived, neutralizing Ab titers against the infecting DV serotype to peak viremia levels measured in subjects with symptomatic dengue. In this subset, we will fit a multiple linear regression model for peak viremia with neutralizing Ab titer levels, and other potential covariates. Model selection will be by partial F-tests [32] on the difference in residual error.

The kinetics of serotype-specific anti-DV neutralizing Abs will be characterized in Filipino infants from 6-14 weeks to 16 months. We will do this using a two stage approach [33]. Serial plasma samples from 30 study subjects who did not have evidence of a DV infection from birth to 16 months, and born to DV-immune mothers (neutralizing Ab titers to $\geq$ 2 DV serotypes), will be collected. In the first stage, the elimination half lives (t½β) of neutralizing Abs to DVs 1-4 will be estimated from the slopes of linear fits to the log Ab titer concentrations over time for each subject. In the second stage, the serotype-specific t½β's will be fitted in linear regression models which will include Ab levels to the other serotypes and other covariates, i.e. factors which might impact maternal Ab decline or otherwise predict Ab levels. The significance of covariates will be assessed using F tests [32]. Model adjusted (least squares or estimated population marginal means [34]) estimates of these parameters with their standard errors will be generated. We will estimate the pre-existing anti-DV neutralizing Ab titers at the time of illness in symptomatic dengue cases using the previously constructed model. Neutralizing Ab titers will be estimated at which 25%, 50% and 75% of the subjects developed a symptomatic DV infection.

II. *Second study objective*- <u>measure the strength of association of anti-DV Ab effects (neutralization or enhancement) with development of DHF, controlling for other potential covariates.</u>

The variables that will be examined as potential covariates were selected to represent different aspects of the infant host response to primary DV infection, and are summarized in the table in Section 7.1. Using the pre-infection samples, potential risk and protective factors will be evaluated in multiple logistic regression analyses. Infants who develop hospitalized DHF will be the disease group (i.e. event), and the other subjects whose disease severity decreases at least one category on the disease severity scale will serve as controls. Models will constructed in a stepwise manner using likelihood ratio chi-squared statistics to evaluate improvement in fit [35]. Adequacy of the fit for the final model will be evaluated using the Hosmer-Lemeshow Goodness of Fit Test [36]. Point and interval estimates of relative risk will be made by exponentiating model coefficients and their 95% confidence intervals [35]. Analyses will be performed with all DV serotypes pooled, and as serotype included as a candidate term in the model.

8 SUBJECT CONFIDENTIALITY

All case report forms will be kept in locked cabinets when not in use. Only one computer database file will have the patient name, residence address, study ID number, or hospital ID number. This file will be kept in secure accounts with limited access requiring a password. All primary and merged computer database files will be kept in secure accounts with password access required. Patients will receive a study ID number identifying the sequence of enrollment. Analytic files will retain only the unique study number attached to each subject in the study. Blood samples will be identified only by different log/specimen numbers when used for immunologic assays and experiments. Only listed study investigators and their personnel will have access to the blood specimens. Banked samples for repeat assays and additional future research studies will be stored under secure conditions at RITM, or at CIDVR with the prior approval of RITM. Parents must consent to future use of stored specimens. Data used for analyses or publication will be based on study number and not contain individual identifying information.

Subject confidentiality is held strictly in trust by the participating investigators, their staff, and the sponsor(s) and their agents. This confidentiality is extended to cover testing of biological samples and genetic tests in addition to the clinical information relating to participating subjects.

The study protocol, documentation, data and all other information generated will be held in strict confidence. No information concerning the study or the data will be released to any unauthorized third party without prior written approval of the sponsor.

The clinical study site will permit access to all documents and records that may require inspection by the sponsor or its authorized representatives, including but not limited to, medical records (office, clinic or hospital) and pharmacy records for the subjects in this study. Sponsor(s) include RITM, UMMS, and NIH/NIAID.

8.1 Future Use of Stored Specimens

Residual blood specimens will be stored at RITM, or at UMMS following RITM permission, after the study is complete. As part of the informed consent process, parents will have the option to allow potential future use of their infant's blood specimens, and mothers will have the option to allow potential future use of their blood specimens. If future use consent is obtained, these blood specimens may be made available to investigators for future studies in a de-identified manner as described above. The protections for confidentiality for any future use of specimens will be as previously described. Study participants may change their mind at any time regarding future use of specimens. If study investigators are notified that consent is withdrawn for future use of specimens, then those samples will be discarded in an appropriate manner after the study-related assays are completed. At the present time, there are no specific plans for future use of blood specimens beyond what is listed in this protocol. Institutional

Review Board approval, or a letter of exemption if applicable, will be required for future use of specimens not specifically listed in this protocol.

9 INFORMED CONSENT PROCESS

The parent/guardian of the infant subject must give written informed consent before being enrolled in this study. There will be separate consent forms for the mother and the infant. Interested parents will be individually counseled regarding the risks and possible benefits of participation in this study. The study protocol and consent forms in Tagalog will be reviewed in detail, questions will be addressed, and written informed consent will be obtained at study visit #1. Up until infant age of 14 weeks, there will be no pre-specified time limit for parents to decide whether they would like their infants to participate. The consent forms in Tagalog will be signed by the parent, witnessed by another person, and signed by study personnel obtaining the informed consent. Participants may withdraw consent and leave the study at any time. Informed consent forms will be provided in duplicate- the original will be kept by the investigator, and a copy will be kept by the subject's parent/guardian. The rights and welfare of subjects will be protected by emphasizing to them that the quality of medical care they receive will not be adversely affected if they decline to participate in this study.

9.1 Informed Consent/Assent Process (in Case of a Minor or Others Unable to Consent for Themselves)

No assent form is necessary for this study.

10 LITERATURE REFERENCES

1. Anonymous, *Dengue and dengue haemorrhagic fever. Fact sheet N.117.* 2002, World Health Organization (WHO): Geneva.

2. Nimmannitya, S., *Dengue hemorrhagic fever: diagnosis and management*, in *Dengue and Dengue Hemorrhagic Fever*, D.J. Gubler and G. Kuno, Editors. 1997, CAB International: New York. p. 133-146.

3. Wills, B.A., et al., *Size and charge characteristics of the protein leak in dengue shock syndrome.* Journal of Infectious Diseases, 2004. **190**: p. 810-818.

4. Sabin, A., *Research on dengue during World War II.* American Journal of Tropical Medicine and Hygiene, 1952. **1**: p. 30-50.

5. Halstead, S.B., *Pathogenesis of dengue: challenges to molecular biology.* Science, 1988. **239**: p. 476-481.

6. Burke, D.S., et al., *A prospective study of dengue infections in Bangkok.* American Journal of Tropical Medicine and Hygiene, 1988. **38**: p. 172-180.

7. Guzman, M.G., et al., *Dengue hemorrhagic fever in Cuba.* American Journal of Tropical Medicine and Hygiene, 1990. **42**: p. 179-184.

8. Halstead, S.B., S. Nimmannitya, and S.N. Cohen, *Observations related to pathogenesis of dengue hemorrhagic fever. IV. Relation of disease severity to antibody response and virus recovered.* Yale Journal of Biology and Medicine, 1970. **42**: p. 311-328.

9. Sangkawibha, N., et al., *Risk factors in dengue shock syndrome: a prospective epidemiological study in Rayong, Thailand. I. The 1980 outbreak.* American Journal of Epidemiology, 1984. **120**: p. 653-669.

10. Thein, S., et al., *Risk factors in dengue shock syndrome.* American Journal of Tropical Medicine and Hygiene, 1997. **56**(5): p. 566-572.

11. Graham, R.R., et al., *A prospective seroepidemiologic study on dengue in children four to nine years of age in Yogyakarta, Indonesia I. Studies in 1995-1996.* American Journal of Tropical Medicine and Hygiene, 1999. **61**(3): p. 412-419.

12. Endy, T.P., et al., *Epidemiology of inapparent and symptomatic acute dengue virus infection: a prospective study of primary school children in Kamphaeng Phet, Thailand.* Am J Epidemiol, 2002. **156**(1): p. 40-51.

13. Endy, T.P., et al., *Relationship of preexisting dengue virus (DV) neutralizing antibody levels to viremia and severity of disease in a prospective cohort study of DV infection in Thailand.* Journal of Infectious Diseases, 2004. **189**: p. 990-1000.

14. Burton, D.R., *Antibodies, viruses, and vaccines.* Nature Reviews Immunology, 2002. **2**: p. 706-13.

15. Halstead, S.B., et al., *Dengue hemorrhagic fever in infants: Research opportunities ignored.* Emerging Infectious Disease, 2002. **8**(12): p. 1474-79.

16. Halstead, S.B., J.S. Porterfield, and E.J. O'Rourke, *Enhancement of dengue virus infection in monocytes by flavivirus antisera.* American Journal of Tropical Medicine and Hygiene, 1980. **29**(4): p. 638-642.

17. Halstead, S.B., *Antibody, macrophages, dengue virus infection, shock, and hemorrhage: a pathogenetic cascade.* Reviews of Infectious Diseases, 1989. **11**(Supplement 4): p. S830-S839.

18. Kliks, S.C., et al., *Evidence that maternal dengue antibodies are important in the development of dengue hemorrhagic fever in infants.* American Journal of Tropical Medicine and Hygiene, 1988. **38**(2): p. 411-19.

19. Anonymous. *Categories of Research that may be reviewed by the IRB through an Expedited Review Procedure*. 1998 [cited; Available from: http://www.hhs.gov/ohrp/humansubjects/guidance/expedited98.htm.

20. Vaughn, D.W., et al., *Dengue viremia titer, antibody response pattern, and virus serotype correlate with disease severity.* Journal of Infectious Diseases, 2000. **181**: p. 2-9.

21. Libraty, D.H., et al., *Differing influences of virus burden and immune activation on disease severity in secondary dengue-3 virus infections.* J Infect Dis, 2002. **185**(9): p. 1213-21.

22. Libraty, D.H., et al., *High circulating levels of the dengue virus nonstructural protein NS1 early in dengue illness correlate with the development of dengue hemorrhagic fever.* J Infect Dis, 2002. **186**(8): p. 1165-8.

23. Lanciotti, R.S., et al., *Rapid detection and typing of dengue viruses from clinical samples by using reverse transcriptase-polymerase chain reaction.* Journal of Clinical Microbiology, 1992. **30**: p. 545-551.

24. Innis, B.L., et al., *An enzyme-linked immunosorbent assay to characterize dengue infections where dengue and Japanese encephalitis co-circulate.* American Journal of Tropical Medicine and Hygiene, 1989. **40**(4): p. 418-427.

25. Clarke, D.H. and J. Casals, *Techniques for hemagglutination and hemagglutination-inhibition with arthropod borne viruses.* American Journal of Tropical Medicine and Hygiene, 1958. **7**: p. 561-573.

26. Goldman, A.S. and R.M. Goldblum, *Transfer of maternal leukocytes to the infant by human milk.* Current Topics in Microbiology Immunology, 1997. **222**: p. 205-13.

27. Jain, L., et al., *In vivo distribution of human milk leucocytes after ingestion by newborn baboons.* Archives of Disease in Childhood, 1989. **64**: p. 930-33.

28. McCullagh, P., *Regression models for ordinal data (with Discussion).* Journal of the Royal Statistical Society, 1980. **42**: p. 109-142.

29. Snell, E.J., *A scaling procedure for ordered categorical data.* Biometrics, 1964. **40**: p. 592-607.

30. Liang, K.-Y. and S.L. Zeger, *Longitudinal data analysis using generalized linear models.* Biometrika, 1986. **73**: p. 13-22.

31. Zeger, S.L., K.-Y. Liang, and P.S. Albert, *Models for longitudinal data: a generalized estimating equation approach.* Biometrics, 1988. **44**: p. 1049-60.

32. Kleinbaum, D.G., L.L. Kupper, and K.E. Muller, *Applied Regression Analysis and Other Multivariable Methods*. 2nd ed. 1988, Boston: PWS-Kent Publishing Co.

33. Anonymous, *Guidance for Industry: Population pharmacokinetics*. 1999, U.S. Dept. of Health and Human Services, Food and Drug Administration: Washington D.C.

34. Searle, S.R., T.P. Speed, and G.A. Milliken, *Population marginal means in the linear model: an alternative to least squares means.* The American Statistician, 1980. **34**: p. 216-221.

35. Hosmer, D.W. and S. Lemeshow, *Applied Logistic Regression*. 1989, New York: John Wiley and Sons, Inc.

36. Hosmer, D.W., et al., *A comparison of goodness-of-fit tests for the logistic regression model.* Stat Med, 1997. **16**(9): p. 965-80.

SUPPLEMENTS/APPENDICES

A. Informed consent form for the infants*.

B. Informed consent form for mothers*.

C. Study advertisement sheet*.

D. Study information for recruitment sessions*

* to be translated into Tagalog.
